# Effective Population Size Predicts Local Rates but Not Local Mitigation of Read-through Errors

**DOI:** 10.1093/molbev/msaa210

**Published:** 2020-08-14

**Authors:** Alexander T Ho, Laurence D Hurst

**Affiliations:** Milner Centre for Evolution, University of Bath, Bath, United Kingdom

**Keywords:** nearly neutral theory, molecular evolution, translational read-through, additional stop codons, phenotypic error, error mitigation

## Abstract

In correctly predicting that selection efficiency is positively correlated with the effective population size (*N*_e_), the nearly neutral theory provides a coherent understanding of between-species variation in numerous genomic parameters, including heritable error (germline mutation) rates. Does the same theory also explain variation in phenotypic error rates and in abundance of error mitigation mechanisms? Translational read-through provides a model to investigate both issues as it is common, mostly nonadaptive, and has good proxy for rate (TAA being the least leaky stop codon) and potential error mitigation via “fail-safe” 3′ additional stop codons (ASCs). Prior theory of translational read-through has suggested that when population sizes are high, weak selection for local mitigation can be effective thus predicting a positive correlation between ASC enrichment and *N*_e_. Contra to prediction, we find that ASC enrichment is not correlated with *N*_e_. ASC enrichment, although highly phylogenetically patchy, is, however, more common both in unicellular species and in genes expressed in unicellular modes in multicellular species. By contrast, *N*_e_ does positively correlate with TAA enrichment. These results imply that local phenotypic error rates, not local mitigation rates, are consistent with a drift barrier/nearly neutral model.

## Introduction

Genomes vary in multiple parameters that typically covary. Some genomes, like ours for example, are “bloated” in the sense that they have large introns, many introns (per bp of coding sequence), large intergene distances, and a high load of transposable elements (TEs) (Lynch and Conery 2003). As a consequence, we have an especially low density of exon to immature transcript size (Warnecke et al. 2008) and a low gene density (Lynch and Conery 2003). Other eukaryotic genomes, such as that of yeast (*Saccharomyces cerevisiae*), are by contrast lithe: Introns are rare and small, intergene distance is low, TE load is relatively light, and gene density is thus high (Lynch and Conery 2003).

How are we to understand not just the variation between genomes in such parameters but also the tendency for multiple measures of genomic “economy” to positively correlate one with the other? Lynch (2007) has forcefully argued that the nearly neutral theory (Ohta 1992) can explain both problems. This proposes that when the effective population size (*N*_e_) is large, selection is relatively efficient at removing deleterious mutations. By contrast when *N*_e_ is low, mutations, such as TE insertions, that would be “seen” as deleterious in species with large *N*_e_ are instead only weakly deleterious or effectively neutral and hence able to be fixed owing to drift. Consistent with this, the level of constraint on protein sequence evolution is higher when *N*_e_ (or proxies to it) is high (Keightley and Eyre-Walker 2000). More particularly, species with low *N*_e_ are expected to accumulate uneconomical sequence, leading to genome bloating. Consistent with this model, intron density and size covary negatively with the effective population size (Lynch and Conery 2003; Wu and Hurst 2015).

The genome bloating concerns what generically might be regarded as aspects of genomic anatomy. Can the same body of theory also explain genomic behavior? Lynch et al. (2016) have also argued that, although the mutation rate is under selection to be as low as possible (Kimura 1967), low *N*_e_ forces a drift barrier preventing especially low mutation rates in species with small *N*_e_. As a consequence, genomes of species with low *N*_e_ have both bloated genomes and high mutation rates (both per bp per generation and especially per genome) (Sung et al. 2012; Lynch et al. 2016).

The mutations considered in these models are one class of error, these being heritable errors. One can also ask about selection on nonheritable (somatic) mutations and nonheritable nonmutational “phenotypic” errors (Burger et al. 2006; Willensdorfer et al. 2007), such as accidental mistranslation, frameshifts, stop-codon read-through, missplicing, misfolding, and so on. Here, we consider between-species variation in phenotypic errors. Errors like these are ubiquitous, occur at high rates, and are typically deleterious (Warnecke and Hurst 2011; Yang et al. 2017; Fu et al. 2018; Liu and Zhang 2018a, 2018b; Li and Zhang 2019).

To resolve these errors, genomes can employ “global” or “local” solutions (Rajon and Masel 2011). Global solutions involve strategies achieved by altering the molecular machinery required for gene expression and hence moderate errors at multiple sites. By contrast, local solutions are employed to ameliorate error at a specific site, or gene. In turn, each class of solution (global/local) can either affect the rate of error or mitigate errors once they have occurred. The pathway to detect and recycle misfolded proteins (Chen et al. 2011; Jackson and Hewitt 2016) may be considered as a global error mitigation device, preventing the buildup of potentially toxic misfolded proteins, whereas employment of chaperones, to direct the correct folding of proteins, may be considered part of a system of global error rate modification, preventing misfolding in the first place. Other examples of global mitigation solutions include improving the machinery required for proofreading during transcription (Zenkin et al. 2006; Gamba and Zenkin 2018) and nonsense-mediated decay (NMD) to trap misspliced transcripts (Kawashima et al. 2009; Tabrez et al. 2017). Further, the genetic code appears to be structured in a manner that reduces the impact of mistranslation (Freeland and Hurst 1998) and, in addition, misacylated tRNAs may tend to mismatch with codons that code for the misloaded amino acids (Seligmann 2011), both of which may be considered as global mitigation strategies.

Here we focus on the problem of selection on local error control devices, in particular to understand how selection on local error rate and local error mitigation vary with *N*_e_. Although it has been suggested that selection for local mutation rate modification (i.e., heritable errors) is too weak (Hodgkinson and Eyre-Walker 2011; Chen and Zhang 2013), even in species with large *N*_e_ (Chen and Zhang 2013), selection on local phenotypic error handling may be different as the underlying rates are higher. As regards local rate modifiers, examples include usage of strong splice sites or exonic splice enhancers (ESEs) to increase splicing accuracy of a specific exon or the use of optimal codons to modify rates of amino acid misincorporation at one codon within a gene (Stoletzki and Eyre-Walker 2007). Numerous local error mitigation mechanisms have also been suggested. Codons mutationally adjacent to stop codons (those one mutation away) are avoided at the 3′-end of human genes (Cusack et al. 2011) where NMD cannot recognize premature stop codons (Zhang et al. 1998). Although this will not affect the rate of mistranscription, it will ensure that mistranscription costs are reduced as especially harmful premature stops codons are less likely to be a consequence of mistranscription. Out-of-frame stop codons are thought to promote translation termination following erroneous frameshift events (Seligmann and Pollock 2004) and have been found to be enriched downstream of frameshift-prone codons (Seligmann 2019). Similarly, in eukaryotes, the presence of in-frame stop codons in introns implies selection to degrade by NMD erroneously spliced mRNA in which introns are retained (Jaillon et al. 2008; Sayani et al. 2008; Brogna and Wen 2009; Ramani et al. 2009; Behringer and Hall 2016). Intronic stop codons occur earlier in the intron than expected by chance, consistent with selection to minimize waste and trigger NMD as soon as possible (Behringer and Hall 2016), although the presence of an in-frame intronic stop codon is no guarantee of NMD-mediated removal on intron retention (Sayani et al. 2008).

The distinction between local error rate and error mitigation control is not always unambiguous. As noted above, selection on codon usage is suggested to alter the rate of amino acid misincorporation (Stoletzki and Eyre-Walker 2007) or mitigate mistranscription events (Cusack et al. 2011). Comparably, in addition to global rate modification, chaperones could in principle mitigate the misfolding effects of mistranscription and mistranslation. Similarly, owing to great enrichment of A at coding site +4, the trinucleotide TGA is greatly enriched at positions 2–4 in bacterial genes (which start NTG) (Abrahams and Hurst 2017). As TGA is a stop codon, this might possibly be to enable rapid frameshift correction, that is, stopping a misaligned ribosome and putting it back into frame by enabling a one base shuttle backward, thereby reducing the net rate of out-of-frame initiation. Alternatively, if the +1-stop codon enables rapid release of the ribosome, it acts to minimize the costs, rather than reducing the rate. Logically it is possible that both occur.

Here we consider what may be a good exemplar for considering relative selection strength on local error rates and local error mitigation, namely the rate and mitigation of translational read-through. Read-through happens when the primary stop codon of an expressed gene is not recognized by its release factor (Roy et al. 2015; Beznoskova et al. 2016) leading to translation of the 3′-untranslated region (UTR) (Doronina and Brown 2006; Namy and Rousset 2010)—see Rodnina et al. (2020) for a recent review. There are some hypothetical advantages of read-though, such as increased proteome diversity (Dunn et al. 2013) and access to additional C-terminal protein domains at low abundance (well described in Pancrustacea [Jungreis et al. 2011], mammals [Eswarappa et al. 2014], yeast [Namy et al. 2003], for example). Read-through may also enable selection to purge deleterious 3′-UTR sequence (Giacomelli et al. 2007; Rajon and Masel 2011; Kosinski and Masel 2020). However, the best evidence suggests that it is typically nonadaptive and arises due to molecular error (Li and Zhang 2019).

The costs of C-terminal extension via read-through have multiple mechanisms. In the absence of a fail-safe stop codon, we might expect degradation of both RNA and nascent protein when the translating ribosome reaches the polyA tail (Dimitrova et al. 2009; Klauer and van Hoof 2012). Should protein be produced following termination at a 3′ fail-safe stop codon there may yet be problems with protein localization (Falini et al. 2005; Hollingsworth and Gross 2013), protein aggregation (Vidal et al. 1999, 2000), and protein stability (Clegg et al. 1971; Namy et al. 2002; Pang et al. 2002; Inada and Aiba 2005; Shibata et al. 2015) causing reduced titer (Arribere et al. 2016). Aside from these, even in a best-case scenario there is likely to be energetic wastage from unnecessary 3′-UTR translation (Wagner 2005).

One reason read-through is a useful exemplar for broad-scale pan taxon analysis is that, unlike the case of splicing error, where different species employ different SR proteins and ESEs and have different intron lengths and densities, the molecular biology of termination is similar across eukaryotes (and to some degree within prokaryotes and archaea) (Capecchi 1967; Grentzmann et al. 1994; Mikuni et al. 1994; Stansfield et al. 1995; Zavialov et al. 2001; Salas-Marco and Bedwell 2004; Alkalaeva et al. 2006; Gao et al. 2007; Dever and Green 2012; Kobayashi et al. 2012).

Furthermore, from genomic analysis alone we can make inferences concerning error rates. This is because both prokaryotes and eukaryotes preferentially use the least leaky stop codon (Strigini and Brickman 1973; Geller and Rich 1980; Parker 1989; Jorgensen et al. 1993; Meng et al. 1995; Sanchez et al. 1998; Tate et al. 1999; Wei et al. 2016; Cridge et al. 2018), TAA, to terminate translation, the preference being strongest where the costs of erroneous read-through would be highest, namely in highly expressed genes (HEGs) (Korkmaz et al. 2014; Trotta 2016). We do not exclude the possibility of other modes of selection acting in favor of TAA. There may, for example, be selection for fast release of the ribosome to prevent ribosomal traffic jams (Tuller et al. 2010). Conserved TAA repeats at specific sites in tRNAs overlapping mRNAs in mtDNA might imply utility beyond its function as a stop codon (Faure and Barthélémy 2019). Furthermore, TAA is robust to two mistranscription events (TAA->TGA, TAG) whereas the two other stop codons are resilient to only one (TGA->TAA, TAG->TAA). We can, however, discern that at least some TAA selection relates to translational read-through by examination of 3′ flanking sequence known to alter read-through rates (Bossi and Roth 1980; Wei and Xia 2017; Cridge et al. 2018). Enrichment of these flanking motifs across genes, aligned with evidence for TAA preference in HEGs, provides solid evidence that read-through is a significant, although not necessarily unique, selection pressure.

A third reason that read-through is a good exemplar is because there is prior evidence for an easy to define error mitigation mechanism. Notably, 3′ in-frame additional stop codons (ASCs) may ameliorate translational error costs by providing a second opportunity (a fail-safe mechanism) to terminate translation (Nichols 1970; Major et al. 2002; Liang et al. 2005; Adachi and Cavalcanti 2009; Fleming and Cavalcanti 2019). ASCs have sometimes been referred to as “tandem stops”; however, we prefer the “ASC” terminology to avoid possible confusion relating to their proximity to the primary stop. The term “tandem stop codon,” for example, sometimes only refers to the immediately proximal in-frame codon position. Similarly, we note that ASCs are distinct from out-of-frame stop codons, these being stops that lie out-of-frame in coding sequence, possibly to ameliorate frameshift errors (Seligmann and Pollock 2004; Abrahams and Hurst 2017). One might expect that selection for ASCs might be stronger the closer they are to the focal stop codon. For this reason, and following prior evidence of enrichment specifically at sites very close to the focal stop (Liang et al. 2005; Adachi and Cavalcanti 2009; Ho and Hurst 2019), we here consider ASC enrichment in the following six in-frame “codon” positions.

Theoretical expectations regarding *N*_e_ and the selection on error rate control and error mitigation are not as simple as stronger selection, and hence greater commonality of both, when *N*_e_ is high (Rajon and Masel 2011; Meer et al. 2020). The situation is especially complex as the global/local and rate/mitigation distinctions provide four mutually dependent axes for selection. Selection on mitigation and rate have the potential to be negatively associated: If rates are low, mitigation is unnecessary; if mitigation is effective, selection on rate reduction diminishes (Rajon and Masel 2011). Similarly, if global error rates are low or global mitigation mechanisms effective, selection for local effects will be weaker and vice versa. These dynamics are even more complicated as correlations can be accentuated by subsequent evolution. If, for example, error read-through rates are low, then 3′ downstream regions are effectively shielded from selection so enabling accumulation of mutations that render read-through more deleterious should it happen, intensifying selection to reduce rates (Rajon and Masel 2011; Meer et al. 2020). This sort of positive feedback loop produces, it is argued, two attractors: mostly deleterious consequences of read-through (no ASCs) coupled with low read-through rates, and mostly benign read-through (owing to ASCs and other devices) coupled with high read-through rates (Rajon and Masel 2011; Meer et al. 2020).

The question then is how the occupancy of these two solutions, assuming these to be the only two stable solutions, might be affected by changes in *N*_e_. Unlike global solutions, local solutions must evolve multiple times in order to affect error handling for multiple genes. Each event must have a low selection coefficient associated with it (as opposed to global modifiers). Considering the case of read-through errors in particular, it was thus argued that a strategy of high error rate with common mitigation is expected under high *N*_e_ (Rajon and Masel 2011; Meer et al. 2020). Conversely as *N*_e_ declines, the solution could shift to globally regulated low error rates (low read-through rates) and absence of mitigation (reduced selection for ASCs).

In support of their model, especially as regards read-through, Rajon and Masel (2011) argue that yeast has a large population size, high read-through error rates with effective local mitigation of read-through, citing previously observed ASC enrichment (Liang et al. 2005). However, there was no comparator from taxa with smaller or larger *N*_e_. Our recent demonstration (Ho and Hurst 2019) that in bacteria (that we presume to mostly have even higher *N*_e_) there is no evidence for ASC enrichment would appear to contradict the prediction of enrichment for local mitigation when *N*_e_ is high (see also Korkmaz et al. [2014]). However, as bacterial and eukaryotic termination mechanisms are not identical the comparison may not be fair.

Meer et al. (2020) argue that the high mistranscription rate in *Escherichia* *coli* compared with species such as *S. cerevisiae* supports the view of higher error rates when *N*_e_ is also high. Meer et al. (2020) also note, however, the possibility of local selection to reduce error rates when *N*_e_ is high as 1) selection is efficient and 2) intrinsic error rates are high. The recognition of the potential relevance of local selection on error rate questions in turn, the assumptions of the original model. The model of Rajon and Masel (2011) assumes that error rate is a globally regulated process associated with trade-offs in translational velocity and growth rate, whereas mitigation is locally regulated (e.g., by selection for ASCs). Local modulation of rate via change in stop codon usage (leaky vs. nonleaky) is not considered. This complicates matters as rate (stop codon choice) and mitigation (ASC selection) are both local variables. As such, both are subject to low selective coefficients and so more likely to be recognized by selection when *N*_e_ is high. In this regard, Rajon and Masel (2011) make no prediction about TAA usage as a function of *N*_e_. However, just as Meer et al. (2020) predict, and observe, lower mistranscription rates in HEGs than in lowly expressed genes (LEGs) when both *N*_e_ and global mistranscription rates are high (e.g., in *E. coli*), so too one can ask whether a greater HEG/LEG TAA disparity is seen when *N*_e_ is high.

Rather than attempting to extend theory to consider the balance between local rate and local mitigation (in a 2 × 2 framework), we shall instead attempt to provide a robust empirical base for theory to address. We shall consider rates of usage of TAA as a local rate reducing modifier and of ASCs as local mitigators of errors. Aside from *N*_e_, however, we also ask about alternative possibly relevant parameters. For example, often when considering error mitigation, a distinction between unicellular and multicellular organisms may be relevant. We presume that any given gene expression error is more threatening to organismal survival in unicellular species compared with multicellular ones. In multicellular species, there are at least two mechanisms through which gene expression fitness effects could be ameliorated. Firstly, low fitness cells generated by molecular error may be removed by apoptosis and subsequently replaced through new cell proliferation and differentiation (Bergmann and Steller 2010; Brock et al. 2019). Secondly, in multicells the reduced productivity of low fitness cells could be ameliorated by the functional redundancy of its neighbors. These avenues are not equally open to all cells within a multicellular species. Indeed, for this sort of reason selection against erroneous protein translation is thought to be more stringent in neurons (Drummond and Wilke 2008). These same avenues for compensation are probably less open to unicell species also. Aside from cellularity, it may be important to consider genome anatomical features such as gene length, intergenic distances, and GC content. As stop codons are GC-poor, GC-rich genomes might be under stronger selection to preserve TAA or ASCs whereas AT-rich genomes have a higher probability of an in-frame ASC by chance. The costs of producing potentially deleterious read-through transcripts might also vary in terms of the proportion of the sequence added or in terms of the absolute length added.

## Results

### Evidence for Selection against Translational Read-through in Eukaryotes

We first sought to strengthen prior evidence (Li and Zhang 2019) that translational read-through is indeed opposed by natural selection in eukaryotes. Given that nucleotides in close downstream proximity to the stop are implicated in stop codon recognition (Bossi and Roth 1980; Cridge et al. 2018; Tate et al. 2018) and hence are under selection to modify translational read-through, we ask 1) whether there is evidence for selective constraint in the vicinity of the stop codon, 2) whether overrepresented motifs reflect selection for read-through suppression specifically, and 3) whether TAA is overrepresented in HEGs. We note that analysis of preferences 5′ of the focal stop codon is complicated by selection on amino acid content that need have little or nothing to do with stop codon recognition. This notion is supported in yeast, 5′ codon usage being uncorrelated with known effects on translation termination (Williams et al. 2004). Indeed, amino acid choice has recently been shown to majorly impact protein expression and decay (Weber et al. 2020). For these reasons, we focus attention on 3′ effects but present 5′ effects for context.

#### Constraint on Substitution Rate Surrounding Stop Codons Is Most Acute Near the Stop Codon

**Fig. 1.**
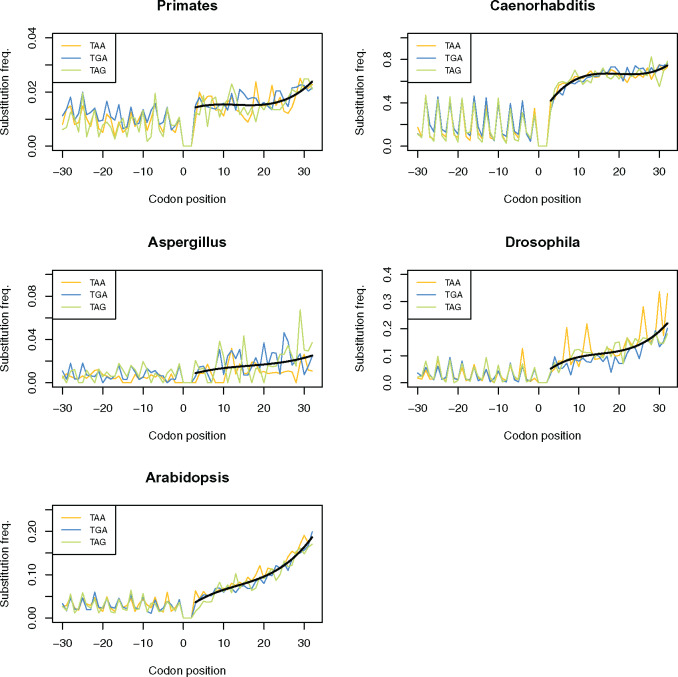
Substitution frequencies of TAA-, TGA-, and TAG-terminating genes at nucleotide positions surrounding the primary stop codon in five eukaryotic groups. Though the profile of change in substitution rate downstream to the stop codon is different between groups, constraint on substitution rate is relieved with increased 3′ distance in TAA-, TGA-, and TAG-terminating genes across all of groups. The black line represents a fitted polynomial line of the average substitution rate across all stop variants.

In bacteria, substitution rate gradually increases with the 3′ distance from the stop codon (Belinky et al. 2018). We here apply the same species triplet method to consider substitution rates surrounding the stop codon in several eukaryotic species (see Materials and Methods). Consistent with the bacterial observations, we find substitution rate to be constrained in close proximity to the primary stop codon in TAA-, TGA-, and TAG-terminating genes across all of our eukaryotic groups (fig. 1). Although the shape of the downstream substitution rate curve is variable between groups, substitution rate is always lowest in close proximity to the stop codon, this being most evident in *Caenorhabditis*, *Drosophila*, and *Arabidopsis* (fig. 1).

#### Preference for Motifs That Decrease Read-through Rates in the Immediate Vicinity of the Stop Codon Is Commonplace in Eukaryotes

**Fig. 2.**
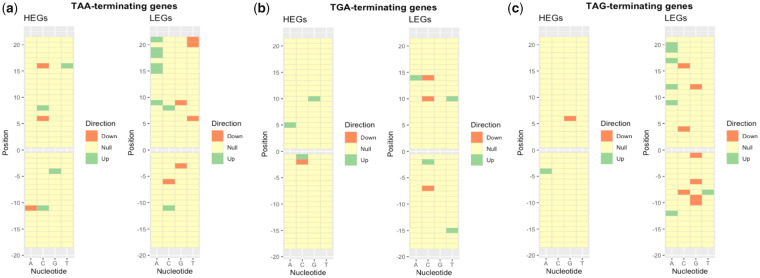
Heat map showing significant nucleotide enrichments and depletions at positions surrounding (*a*) TAA, (*b*) TGA, and (*c*) TAG stop codons in highly expressed human genes. Significant enrichments and depletions in HEGs were determined by chi-square tests (*P* < 0.05) relative to a null expectation from all genes (regardless of expression level).

Constraint on substitution rate alone, however, need not be evidence for selection against translational read-through. Stop codon recognition is not the sole function of the 3′-UTR sequence, these sequences also containing regulatory motifs, binding sites for translational regulators, and so on (Kuersten and Goodwin 2003; Mayr 2019). To ascertain whether substitution constraint was attributable to selection for read-through modifying motifs, we assessed the sequence surrounding stop codons in HEGs for significant nucleotide enrichments and depletions (compared with global levels; fig. 2) and ask whether they relate to known read-through modulators (Cridge et al. 2018). Looking for enrichment in HEGs (compared with all genes) allows us to focus our analysis on identifying motifs that may decrease read-through, under the assumption that these genes are where the costs of aberrant stop recognition are the most extreme.

We find that certain site-specific nucleotide enrichments (*P* < 0.05) are consistent (>3 genomes; supplementary table T1 for TAA-terminating genes, supplementary table T2 for TGA-terminating genes, supplementary table T3 for TAG-terminating genes, Supplementary Material online) among eukaryotes. The observed trends are also consistent with selection to mitigate read-through. Notably, many of these common nucleotide preferences (e.g., +4G or +5C following TAA) have previously been experimentally determined to decrease read-through rate (Cridge et al. 2018). We hence conclude that translational read-though is indeed a significant error in gene expression that triggers local error rate selection on 3′ sequence in response.

#### TAA Stop Codons Are More Strongly Preferred in Highly Expressed Eukaryotic Genes

Having established that read-though is a significant selection pressure, we next assess whether TAA enrichment is a common evolutionary response. The assumption that TAA is the least leaky stop predicts that TAA stops should be the most common across all genomes, all else being equal. However, all else is not equal, the most common stop for any given genome being well predicted by GC content which is highly variable between species (supplementary fig. S1, Supplementary Material online). As expected if there is some form of GC pressure, the relative usage of TAA is negatively correlated with GC content (*P* = 3.2 × 10^−6^, rho = −0.803; Spearman’s rank), with TGA (*P* = 0.00083, rho = 0.636; Spearman’s rank) and TAG (*P* = 0.00012, rho = 0.705; Spearman’s rank) positively correlated. Similar to the trends observed in bacteria previously (Korkmaz et al. 2014), TAG is universally unfavored despite its identical nucleotide composition to TGA.

**Fig. 3.**
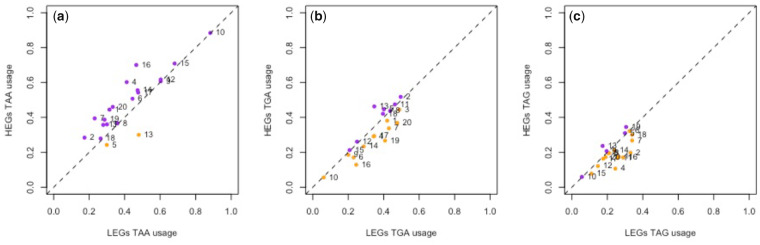
Difference in the usage of (*a*) TAA, (*b*) TGA, and (*c*) TAG stop codons between HEGs and LEGs in 20 eukaryotic species. HEGs are the top quartile of genes expressed according to experimentally derived protein abundance data. LEGs are defined as the bottom quartile of expressed genes. The dotted line in each plot represents equal codon usage between HEGs and LEGs, hence points above the line represent overusage in HEGs (colored purple) and points under the line represent overusage in LEGs (colored orange). In our sample, 18/20 genomes contain higher TAA frequency in HEGs compared with LEGs. Numbered data points correspond to the following species: 1, *Gallus gallus*; 2, *Bos taurus*; 3, *Homo sapiens*; 4, *Xenopus tropicalis*; 5, *Aspergillus niger*; 6, *Drosophila melanogaster*; 7, *Chlamydomonas reinhardtii*; 8, *Arabidopsis thaliana*; 9, *Schizosaccharomyces pombe*; 10, *Dictyostelium discoideum*; 11, *Equus caballus*; 12, *Apis mellifera*; 13, *Rattus norvegicus*; 14, *Saccharomyces cerevisiae*; 15, *Plasmodium falciparum*; 16, *Anopheles gambiae*; 17, *Caenorhabditis elegans*; 18, *Oryza sativa*; 19, *Trypanosoma brucei*; 20, *Danio rerio*.

Given the above, rather than simply considering raw TAA usage between genomes, a fairer way to address whether there might be selection favoring TAA is to ask whether TAA is preferred in HEGs compared with LEGs within the same genome, expression level being a key modifier in the evolutionary dynamics of local error traps (Xiong et al. 2017). Consistent with TAA selection, across a data set of 20 species (15 multicellular and 5 unicellular) for which we have proteomic data we find 18/20 possess higher TAA usage in HEGs (fig. 3). This significantly exceeds the simplest null expectation of a 50:50 split of TAA preference between HEGs and LEGs (*P* = 0.0002, one-tailed binomial test with null *P* = 0.5). Moreover, in HEGs, the observed TAA stop frequencies across our species are significantly higher than those of TGA (*P* = 0.0047; Wilcoxon signed-rank test) and TAG (*P* = 1.33 × 10^−8^; Wilcoxon signed-rank test) in the same species. This contrasts with what is seen in LEGs, where we recover no significant difference between TAA and TGA frequency across our data set (*P* = 0.29; Wilcoxon signed-rank test). In LEGs, TAA frequencies are, however, higher than TAG (*P* = 0.00029; Wilcoxon signed-rank test) possibly reflecting the fact that TAG is the leakiest stop and least favored.

### ASCs Are Enriched in HEGs Predominantly in Genomes Where ASCs Are Globally Enriched

**Fig. 4.**
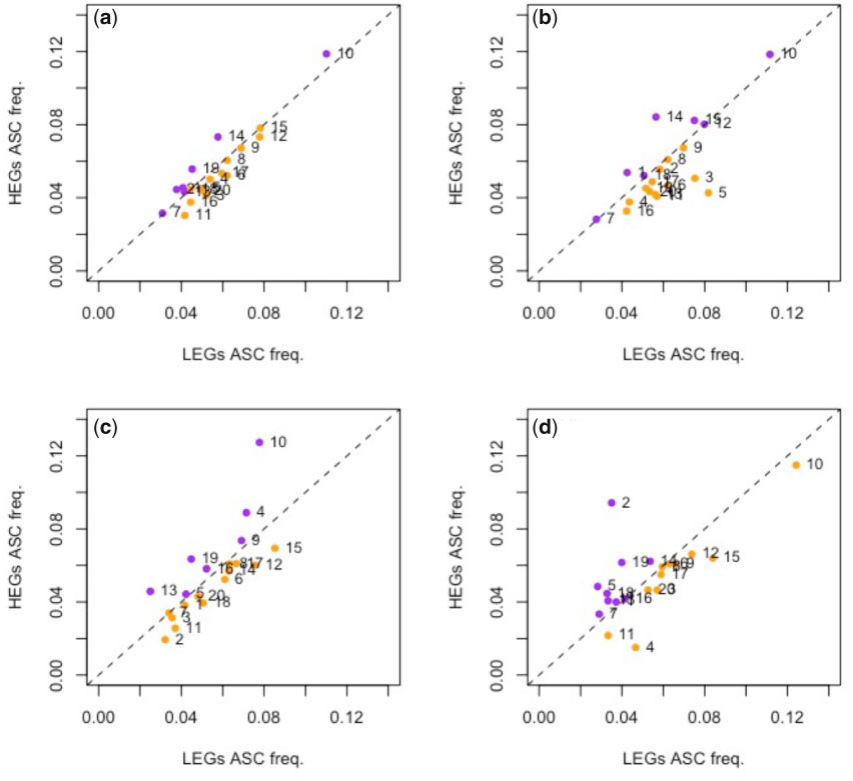
Difference in ASC frequency between genes of high and low expression across (*a*) all genes, (*b*) TAA-terminating genes, (*c*) TGA-terminating genes, and (*d*) TAG-terminating genes in 20 eukaryotic species. HEGs are the top quartile of genes expressed according to experimentally derived protein abundance data. LEGs are defined as the bottom quartile of expressed genes. The dotted line in each plot represents equal ASC frequency in HEGs and LEGs, hence points above the line represent overusage in HEGs (colored purple) and points under the line represent overusage in LEGs (colored orange). In our sample, 7/20 genomes contain higher ASC frequency in HEGs compared with LEGs when considering all genes. Numbered data points correspond to the following species: 1, *Gallus gallus*; 2, *Bos taurus*; 3, *Homo sapiens*; 4, *Xenopus tropicalis*; 5, *Aspergillus niger*; 6, *Drosophila melanogaster*; 7, *Chlamydomonas reinhardtii*; 8, *Arabidopsis thaliana*; 9, *Schizosaccharomyces pombe*; 10, *Dictyostelium discoideum*; 11, *Equus caballus*; 12, *Apis mellifera*; 13, *Rattus norvegicus*; 14, *Saccharomyces cerevisiae*; 15, *Plasmodium falciparum*; 16, *Anopheles gambiae*; 17, *Caenorhabditis elegans*; 18, *Oryza sativa*; 19, *Trypanosoma brucei*; 20, *Danio rerio*.

As with TAA stop codons we can also ask whether ASCs in the first six in-frame codon positions are preferred in HEGs (fig. 4), well-described ASC enrichment being previously witnessed in such proximity to the focal stop (Nichols 1970; Major et al. 2002; Liang et al. 2005; Adachi and Cavalcanti 2009; Fleming and Cavalcanti 2019; Ho and Hurst 2019). Using the same data set, we find that only 7/20 genomes possess an excess of ASCs in HEGs compared with LEGs when considering genes that end in any stop. This is no different than expected under the 50:50 null (*P* = 0.26, two-tailed binomial test with null *P* = 0.5). This might, however, be complicated by the fact that TAA-ending genes are also less leaky and highly expressed. However, we do not observe any deviation from this null across any of the primary stop groups either (7/20 genomes when considering TAA-terminating genes, 7/20 considering TGA-terminating genes, 9/20 considering TAG-terminating genes, all results *P* > 0.05, two-tailed binomial tests with null *P* = 0.5).

Although prima facie the above suggests that no selection is acting upon ASCs, we instead suggest that this owes to the phylogenetic patchiness of ASC enrichment. Across vertebrates, plants, fungi, and invertebrates we find some genomes that have significant ASC enrichment, and others that do not (supplementary fig. S2, Supplementary Material online), suggesting that strong ASC selection is not common to all species, but particular to a few. Specifically, we see no evidence for such enrichment in vertebrates as a group, but more species show enrichment in plants, fungi, and invertebrates than expected by chance (supplementary fig. S2, Supplementary Material online). If only some genomes, for whatever reason, have selection for ASCs these should be enriched for genomes showing an excess in HEGs compared with LEGs. Indeed, of the seven genomes that possess an excess of ASCs in HEGs compared with LEGs (considering all genes), four contain significant ASC enrichment. By contrast, only one genome contains significant ASC enrichment out of the 13 which have ASC excess in LEGs compared with HEGs. These proportions are significantly different (*P* = 0.031, Fisher’s exact test), suggesting that if ASCs are under selection it is in HEGs that they are most common. However, the same result also suggests that in many species ASCs are not under strong selection.

### *N*_e_ Predicts TAA Usage, but Not ASC Enrichment

As we reported previously (Ho and Hurst 2019), some but not all unicellular eukaryotes show evidence of statistically significant ASC enrichment. As described above, this patchiness of ASC enrichment is observed in multicellular eukaryotes too. We note that in all multicellular groups we have analyzed, ASC enrichment is rarer than seen in unicellular species (supplementary fig. S3, Supplementary Material online). Similarly, pooling all multicellular HEGs together and all unicell HEGs together we find TAA usage in unicells (62.7%) to significantly exceed that of multicells (43.7%) (*P *< 2.2 × 10^−16^, χ2 = 597.1, chi-square test 1 df). Such variation within groups, and between unicells and multicells, could potentially be explained by *N*_e_ or cellularity but our analysis has so far failed to control for phylogeny.

To test for correlation between TAA enrichment and *N*_e_, we gather a sample of species for which we have an *N*_e_ estimate. As this sample contained a few species pairs that are especially phylogenetically close (and thus especially influential in the face of parameter estimation error), we pruned the species sample (and phylogenetic tree) to remove closely related species pairs with low species divergence times, leaving one of the two (e.g., human–chimp was resolved to human alone) (supplementary fig. S4, Supplementary Material online). For each genome in our reduced species list (*n* = 15), we calculate a TAA enrichment score taking into account background nucleotide usage (see Materials and Methods) and compare this parameter with *N*_e_ in phylogenetically controlled regression analyses (using phylogenetic generelized least squares [PGLS] tests). We find robust evidence to support a positive relationship between *N*_e_ and TAA enrichment (adjusted *r*^2^ = 0.55, *P* = 0.00098, λ = 0.0; PGLS).

To test the comparable prediction for ASC enrichment, we calculated an ASC enrichment score for each genome. Interestingly, although a phylogenetically uncontrolled analysis reports significance in the direction expected (supplementary fig. S5, Supplementary Material online), a significant relationship between ASC enrichment and *N*_e_ was not recovered (adjusted *r*^2^ = −0.07, *P* = 0.85, λ = 1.0; PGLS). This is because ASC enrichment shows a high rate of phylogenetic autocorrelation (*P* = 0.03 for λ = 0.0, *P* = 1.0 for λ = 1.0), the high λ value suggesting that the trait is evolving as expected given the tree topology alone.

These results suggest that, although TAA enrichment and ASC enrichment are both adaptations to translational read-through, TAA usage is consistent with expectations from the nearly neutral theory but ASC enrichment is not. Instead, its distribution appears to be patchy.

### No Significant Correlation between *N*_e_ and TAA HEG/LEG Disparity

Meer et al. (2020) note that when *N*_e_ is high, there is a greater disparity in mistranscriptional error rates between HEGs and LEGs than there is when *N*_e_ is low. Here, we ask whether there is similarly a greater HEG/LEG TAA disparity when *N*_e_ is high. To test this, we employ protein abundance data to identify HEGs and LEGs for the species in our tree (due to lack of available data, we are reduced to *n* = 11). The variable to be measured for association with *N*_e_ was TAA frequency in HEGs divided by TAA frequency in LEGs (that we call TAA disparity). We find no significant relationship between TAA disparity and *N*_e_ in phylogenetic-controlled analysis (*P* > 0.05) (adjusted *r*^2^ = 0.19, *P* = 0.10, λ = 0.0; PGLS). We note too that the effect size measured by adjusted *r*^2^ at 0.19 is substantially lower than that observed for the TAA enrichment–*N*_e_ effect (adjusted *r*^2^ = 0.55). One possible caveat, however, is that the sample size here is a little lower than in the *N*_e_–TAA enrichment correlation (*n* = 11 and *n* = 15) and λ is low indicating that phylogeny alone cannot explain all of the data. However, using the same species as in the TAA analysis (i.e., with *n* = 11), *N*_e_ remains strongly and significantly correlated with TAA enrichment (see above, and Materials and Methods) (adjusted *r*^2^ = 0.39, *P* = 0.024, λ = 0.0; PGLS). This suggests that we have enough statistical power to detect a correlation between HEG/LEG TAA disparity and *N*_e_, at least if there was one of the same magnitude as seen with TAA enrichment.

### Cellularity Predicts ASC Enrichment but Not TAA Usage

The results described so far suggest that high *N*_e_ genomes favor the most effective stop codon, especially in HEGs. However, *N*_e_ appears to have no ability to predict between-species variation in ASCs, at least after phylogenetic control. What might then explain such variation? We ask whether cellularity may be a predictor as multicellularity may protect against gene expression error by either cell redundancy or cell replacement.

First, we ask whether cellularity (considered as a binary trait in PGLS analysis using the same species tree) predicts TAA and ASC enrichment. We find that it does not for TAA enrichment (adjusted *r*^2^ = 0.022, *P* = 0.27, λ = 0.0; PGLS) but does for ASC enrichment (table 1). This test, although suggestive of a role for cellularity in prediction of ASCs, could be criticized as it overlooks the possible interaction between the TAA and ASCs, namely a gene with TAA may not require ASCs, although in yeast ASCs are used most commonly when associated with TAA (Liang et al. 2005). To consider this issue we divide genes into TAA ending and non-TAA ending (table 1). We find that the connection between cellularity and ASC usage is unaffected. However, there emerges the possibility of ASC enrichment in non-TAA-ending genes being predicted by *N*_e_, although this is sensitive to Bonferroni correction (at *P *< 0.05/3).

**Table 1.** ASC Enrichment Scores Assessed for a Relationship with *N*_e_ or Cellularity by Linear Regression Using PGLS.

Dependent Variable	Gene Set	Ne	Cellularity
ASC enrichment	All genes	*P* = 0.85, *r*^2^ = –0.07	*P* = 0.0021, *r*^2^ = 0.49
	Non-TAA-ending genes	*P* = 0.041, *r*^2^ = 0.23	*P* = 0.0024, *r*^2^ = 0.48
	TAA-ending genes	*P* = 0.98, *r*^2^ = –0.08	*P* = 0.0026, *r*^2^ = 0.48

Note.—ASC enrichment score was calculated for three different sets of genes for each eukaryotic genome in our data set: all genes, non-TAA-ending genes, TAA-ending genes. The resultant scores were then assessed for a relationship with either *N*_e_ or cellularity in a phylogenetically controlled manner. *P*-values and *r*^2^-values are given for each scenario. *r*^2^-values given are the adjusted *r*^2^-values, hence why some are negative.

As cellularity and *N*_e_ are likely to covary, the further (and possibly fairer) comparison is to consider the ASC and TAA enrichment jointly by both cellularity and *N*_e_ (table 2). This we do using a multiple regression model within PGLS. The resulting model, using the ASC enrichment scores calculated from all genes, has a significant fit to the data (adjusted *r*^2^ = 0.45, *P* = 0.011, λ = 0.0; multiple regression PGLS) with cellularity remaining a significant predictor (*P* = 0.015), unlike *N*_e_ (*P* = 0.77). The presence of a significant relationship for cellularity, but not *N*_e_, with ASC enrichment is also evident both when we restrict our gene sets to non-TAA- and TAA-ending genes. Notably, the earlier observed weak correlation between ASC enrichment and *N*_e_ when TAA genes are excluded is removed upon control for cellularity. The same multiple regression method for TAA enrichment finds, as before, that *N*_e_ is a significant predictor (*P* = 0.0018), unlike cellularity (*P* = 0.37).

**Table 2.** ASC Enrichment Scores Assessed for a Relationship with *N*_e_ or Cellularity by Including Both Parameters in Multiple Regression PGLS.

Dependent Variable	Gene Set	*N*_e_	Cellularity	Adjusted *r*^2^
ASC enrichment	All genes	*P* = 0.77	*P* = 0.015	*r* ^2^ = 0.45
	Non-TAA-ending genes	*P* = 0.25	*P* = 0.032	*r* ^2^ = 0.50
	TAA-ending genes	*P* = 0.12	*P* = 0.0013	*r* ^2^ = 0.54

Note.—ASC enrichment score was calculated for three different sets of genes for each eukaryotic genome in our data set: all genes, non-TAA-ending genes, TAA-ending genes. The resultant scores were then assessed for a relationship with either *N*_e_ or cellularity in a phylogenetically controlled multiple regression. *P*-values are given for each coefficient and the adjusted *r*^2^-value is reported for each overall model.

These results suggest that *N*_e_, but not cellularity, predicts the usage of the least leaky stop codon, consistent with classical nearly neutral theory. By contrast, enrichment of ASCs is predicted by cellularity and not by *N*_e_, the latter being contrary to the predictions of Rajon and Masel (2011).

### GC Content May Play a Minor Role in TAA Enrichment

Aside from *N*_e_ and cellularity, it is possible that genome architecture plays a role in both TAA and ASC selection. Might such factors help explain the patchiness of ASC enrichment across species of the same taxonomic group or cellular state? For example, as stop codons are AT-rich, GC-rich genomes contain fewer TAA stops and 3′ ASCs by chance and hence might be under higher selection pressure to preserve existing ones. Additionally, shorter average gene size might modulate the intensity of selection, possibly because the costs associated with the misprocessing of long genes are higher owing to greater wastage. Larger average 3′ intergenic distance may also ensure that TAA primary stops and ASCs are under stronger selection in order to minimize the amount of misprocessing following stop codon read-through.

Considering all three variables in a multiple regression, we find GC content to be the lone significant coefficient when predicting TAA enrichment (*P* = 0.028) despite the overall model having a near significant fit to the data (adjusted *r*^2^ = 0.30, *P* = 0.075, λ = 0.0; PGLS). This relationship is positive, consistent with the view that TAA stops are increasingly preserved in GC-rich genomes. Using the same methodology, ASC enrichment is not predicted by GC content (*P* = 0.99), median gene body length (*P* = 0.53) or median 3′ intergenic distance (*P* = 0.52), the overall model being a nonsignificant fit to the data (adjusted *r*^2^ = −0.18, *P* = 0.83, λ = 1.0; PGLS).

The above results suggest the most relevant model, at least for TAA usage, may be one in which GC, cellularity, and *N*_e_ are employed as predictors. In such a model, GC content does not remain a significant predictor of TAA usage (overall model: adjusted *r*^2^ = 0.60, *P* = 0.0042, λ = 0.0; *N*_e_: *P* = 0.013; cellularity: *P* = 0.97; GC: *P* = 0.13; PGLS). The same model still finds that cellularity (*P* = 0.028), but neither *N*_e_ (*P* = 0.96) nor GC (*P* = 0.69), is a predictor of ASC enrichment (overall model: adjusted *r*^2^ = 0.41, *P* = 0.031, λ = 0.0; PGLS).

We conclude that GC content plays no more than a minor role in TAA selection at the primary site and that genome architecture is otherwise unimportant in the identification of genome-wide TAA and ASC enrichment.

### Marginal Evidence That Genes Associated with Expression in Unicell Mode Contain More ASCs Than Genes Associated with Multicellular Expression in the Same Organism

The above analyses suggest a role for *N*_e_ alone in determining the usage of error-preventing TAA, whereas impact mitigating ASCs were predicted by cellularity. The latter, being a novel result, merits further consideration. Indeed, it would be helpful to have a further means to test the cellularity model controlling for *N*_e_. We suggest that this could be achieved by comparing genes expressed exclusively in the unicell mode with those expressed exclusively in the multicell mode in the same species. We consider two such comparisons: between pollen-specific genes and genes expressed more often in the whole plant body (for brevity, pollen-reduced genes) in *Arabidopsis thaliana* and between the unicellular free-living amoeboid phase and the multicellular phase in the cellular slime mold *Dictyostelium discoideum*. Neither comparison is perfect but to some extent as a pair they control for each other’s weaknesses. In *A. thaliana* we are, for example, comparing common multicellular expression with rare single cell expression, whereas in *D. discoideum* the unicell mode of expression is the common mode of gene expression. However, in *Arabidopsis* we also have a difference between haploid and diploid expression which is uncontrolled.

#### Dictyostelium discoideum *Unicell-Expressed Genes Have an Excess of +1 ASCs*

**Fig. 5.**
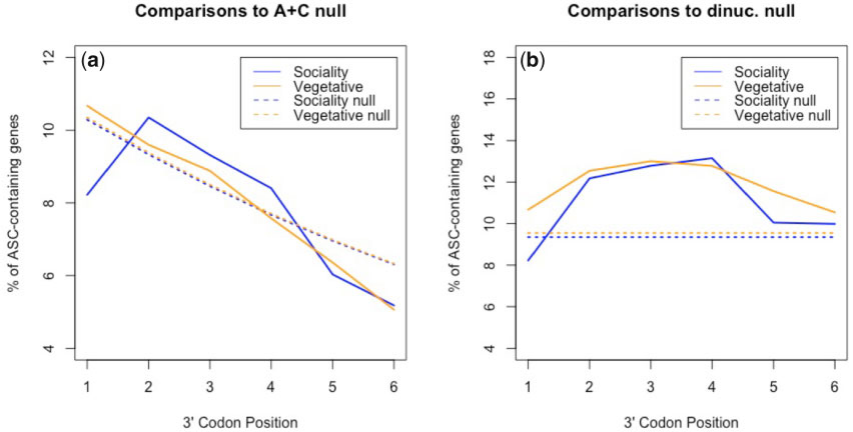
Assessment of ASC enrichment against (*a*) the Adachi & Cavalcanti null and (*b*) dinucleotide-controlled simulations in the 3′-UTRs of sociality- and vegetative-growth associated genes in *Dictyostelium discoideum*. ASC frequencies in vegetative stage expressed genes are enriched (*P* < 0.001; chi-square tests with 1 df) across all positions compared with the dinucleotide null. Against the same null ASCs in sociality genes are enriched at positions +2 to +4 (*P* < 0.001; chi-square tests with 1 df), suggesting that these are the most optimum locations for ASC enrichment within *D. discoideum* within our chosen UTR range. Against the A+C null, vegetative gene ASC frequency is nondeviant (*P* = 0.32; chi-square test with 1 df) whereas sociality gene ASC frequency is significantly lower than expected (*P* = 0.0089, chi-square test with 1 df).

The cellularity hypothesis predicts ASCs to be enriched in vegetative (single cell) expressed genes compared with sociality (multicellular) genes. Considering all six 3′ codon positions together this is observed (χ^2^ = 4.76, *P* = 0.029; chi-square test with 1 df). Examined on a site-by-site basis, ASCs are significantly enriched in vegetative genes compared with sociality genes at position +1 (*P* = 0.0035, chi-square test with 1 df), but no other position within our chosen UTR range (positions +2 to +6: *P* > 0.05). Although there is no significant difference in ASC frequency between vegetative and social genes across positions +2 to +6, ASC frequencies in vegetative stage expressed genes are nonetheless strongly enriched (*P *< 0.001; chi-square tests with 1 df; fig. 5) across all positions compared with dinucleotide-controlled null. ASCs in sociality genes are also enriched beyond dinucleotide expectations at positions +2 to +4 (*P *< 0.001; chi-square tests with 1 df), suggesting that these are the most optimum locations for ASC enrichment within the species within our chosen UTR range. The position +1 difference between vegetative and sociality genes can also be observed when comparing genes against the Adachi and Cavalcanti (2009) null (see Materials and Methods), vegetative gene ASC frequency being nondeviant (*P* = 0.32; chi-square test with 1 df) and sociality gene ASC frequency being significantly lower than expected (*P* = 0.0089, chi-square test with 1 df). This difference is not only consistent with our cellularity prediction but also prima facie consistent with the possible prediction of the fail-safe hypothesis that ASCs should be most strongly selected immediately after the primary stop codon to minimize the error made following read-through.

Might this effect alternatively be owing to a more general thymine nucleotide preference (+4T) following the primary stop that affects position +1 ASC frequency, as seen in bacteria (Major et al. 2002; Wei and Xia 2017)? Contra to this possibility, we find T-starting codons (excluding TGA, TAA, and TAG) to be significantly enriched in sociality genes rather than vegetative genes at this site (*P *< 0.0001, chi-square test with 1 df). This is the opposite to what would be expected if +4T enrichment were to explain the ASC difference observed between vegetative and social genes.

#### Arabidopsis thaliana *Unicell-Expressed Genes Have an Excess of +1 ASCs*

Per the cellularity hypothesis, we predict pollen-specific genes to be more likely to contain ASCs than pollen-reduced genes, in spite of them being less expressed. However, UTR-wide ASC frequency (all positions +1 to +6) is not significantly deviant between the two gene sets (χ^2^ = 1.33, *P* = 0.25; chi-square test with 1 df). Considering each position in isolation, we find that ASCs in pollen-specific genes are, however, significantly enriched compared with pollen-reduced genes at position +1 (*P* = 0.015, chi-square test with 1 df), consistent with prior evidence for ASC selection at this site in *A. thaliana* (Kochetov et al. 2011). There is no significant difference at any other position within our chosen 3′-UTR range (pos +2: *P* = 0.39, pos +3: *P* = 0.90, pos +4: *P* = 0.56, pos +5: *P* = 0.87, pos +6: *P* = 0.93). Again, we acknowledge the possibility that the position +1 ASC difference occurs due to nucleotide preference in proximity to the primary stop. We reject this possibility, finding no significant difference in T-starting codon (excluding TAA, TGA, and TAG) frequency at position +1 between pollen-specific and pollen-reduced genes (χ^2^ = 1.7, *P* = 0.20; chi-square test with 1 df).

Does this result truly reflect a difference between unicell and multicell expressed genes or might the signal observed merely represent a difference between plant tissues, irrespective of cellularity? We can test this by comparing multicellular tissues. Consistent with there being no difference between tissues of the same state, UTR-wide ASC frequencies between leaf-specific and non-leaf-specific genes are nondeviant (χ^2^ = 0.24, *P* = 0.63; chi-square test with 1 df). Taking each position in isolation, there are no differences anywhere between positions +1 to +6 (*P* > 0.05). Similarly, comparing silique-specific genes to non-silique-specific genes also finds no evidence to support deviant ASC frequencies (UTR-wide and all positional results *P* > 0.05; chi-square tests with 1 df).

**Fig. 6.**
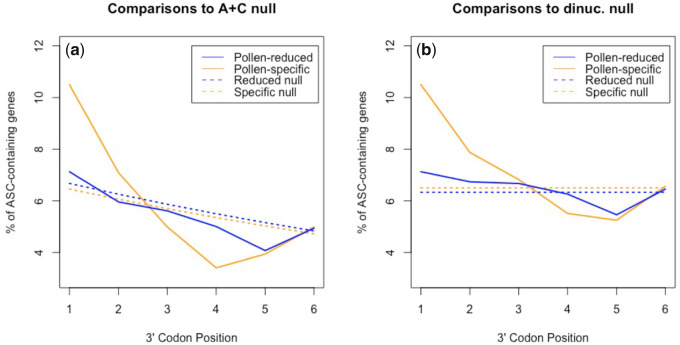
Assessment of ASC enrichment against (*a*) the Adachi & Cavalcanti null and (*b*) dinucleotide-controlled simulations in the 3′-UTRs of pollen-specific and pollen-reduced genes in *Arabidopsis thaliana*. Against dinucleotide-controlled simulations, the ASC frequencies of both sets of genes are significantly enriched at position +1 (pollen-specific genes: *P* = 0.0022, pollen-reduced genes: *P* = 0.031, chi-square tests with 1 df). Consistent with the cellularity hypothesis, significance is an order of magnitude weaker in the case of pollen-reduced genes. When compared with the A+C null, there is evidence of ASC enrichment at position +1 (*P* = 0.0019, chi-square test with 1 df) in pollen-specific but not pollen-reduced genes (*P* = 0.23, chi-square test with 1 df).

In our analysis of ASC enrichment in multicellular species (supplementary fig. S1, Supplementary Material online), we detected significant ASC enrichment at position +1 in *A. thaliana*. Is this still the case in pollen-reduced genes that are rarely expressed in the unicell mode or might the trend be predominantly owing to the pollen-expressed genes? To assess this, pollen-specific and pollen-reduced genes were compared with both dinucleotide-controlled and A+C null at position +1. Against dinucleotide-controlled simulations, the ASC frequencies of both sets of genes are significantly enriched at this position (pollen-specific genes: *P* = 0.0022, pollen-reduced genes: *P* = 0.031, chi-square tests with 1 df; fig. 6). However, when compared with the A+C null, there is evidence of ASC enrichment at position +1 (*P* = 0.0019, chi-square test with 1 df) in pollen-specific but not pollen-reduced genes (*P* = 0.23, chi-square test with 1 df). The pollen case study hence concurs with our evidence that unicellularity may play some role in determining selection for error mitigation.

#### Weak Evidence for TAA Enrichment in Single-Cell Expressed Genes

Above, we have considered ASC usage as a function of cellularity within the same species. If the prior results comparing between species hold, we do not expect to see much, if any, evidence for TAA enrichment in the single-cell phase. In the slime mold, TAA is found at a slightly higher frequency in vegetative genes (89.5%) than multicell genes (87.7%) (χ^2^ = 4.4, *P* = 0.036; chi-square test with 1 df). In *Arabidopsis*, TAA usage is higher in absolute terms in pollen-specific genes (40.1% of genes, compared with 36.0% in pollen-reduced genes) but not significantly so (χ^2^ = 2.5, *P* = 0.12; chi-square test with 1 df). We suggest that these present weak support at best for a coupling between cellularity and TAA usage.

## Discussion

When considering the evolution of the rate of heritable errors (i.e., mutations), the drift-barrier model for the evolution of the mutation rate (Sung et al. 2012; Lynch et al. 2016) proposes that heritable error rates will be higher when *N*_e_ is lower. By contrast, Rajon and Masel (2011) suggest that in species of large effective population size (*N*_e_) there is more effective selection favoring local error mitigation (i.e., more ASCs) hence relaxing selection on global rate modification. We find that the prediction of Rajon and Masel (2011) for ASC enrichment to be higher when *N*_e_ is high to not be supported. We also find that their further result of greater HEG/LEG disparity when *N*_e_ is high (Meer et al. 2020) is not replicated as regards selection for TAA.

By contrast, we do find that TAA preference, and hence local error rate, is predicted by *N*_e_ although whether absolute global rates also vary with *N*_e_ we cannot address. This conclusion assumes that TAA, the preferred stop codon in HEGs in nearly all of our species, because it is associated with lower read-through rates. We showed both enrichment of motifs associated with reduced read-through in HEGs and a general preference for TAA in HEGs, both indicative of selection on TAA to enable low read-through rates. It would be valuable to empirically test this prediction across multiple eukaryotic species, but it is promising that available experimental data are consistent (Cridge et al. 2018). With this caveat, we suggest therefore that our results provide prima facie support for the drift-barrier model being applied to understand the fate of mutations affecting the rate of local phenotypic errors.

### Why Might the Prior Model Be Wrong?

Rajon and Masel (2011) predict higher ASC usage when *N*_e_ is high. This we did not observe. Why might the model of Rajon and Masel (2011) be wrong? We consider several possibilities. Firstly, this may reflect the fact that selection on ASCs is but one mode of locally selected mitigation on read-through. In yeast, potential C-terminal extensions may become preadapted for read-through events (evidenced by higher intrinsic structural disorder) (Kosinski and Masel 2020). In mammalian cells, increased hydrophobicity in 3′-UTR encoded sequence has been linked to more efficient translation arrest when termination fails (Hashimoto et al. 2019). However, unless the relative importance of ASCs as a mode of local mitigation itself varies with *N*_e_, there is no reason to suppose that the prediction of Rajon and Masel (2011), that they supported by reference to selection on ASCs specifically, is incorrect.

Secondly, might our analysis be too conservative? That we find *N*_e_ to predict ASC enrichment in phylogenetically uncontrolled tests is a provocative result given the use of phylogenetic control in similar studies has been contentious. For example, the associations between genome complexity and *N*_e_ described by Lynch and Conery (2003) were observed without control for phylogeny and were subsequently found to not be robust to phylogenetic control (Whitney and Garland 2010). However, more recently, a relationship between *N*_e_ and intron size/number has been recovered using PGLS, albeit with more data points and more recent *N*_e_ estimates (Wu and Hurst 2015). On balance it seems that the most stringent tests are those that are phylogenetically controlled and hence, to err on the side of caution, we prefer the argument that there is no link between *N*_e_ and ASCs. However, given the findings of Wu and Hurst (2015), we acknowledge the possibility that the lack of observed relationship between these two variables may not be resilient to improved sample size and improved *N*_e_ estimation. We note too that *N*_e_ estimation makes an assumption that the populations are at equilibrium which need not be true but should just factor as a noise variable in the analysis. Nevertheless, the association between *N*_e_ and ASC enrichment must be, at the very least, weaker than that observed between *N*_e_ and TAA enrichment given that we find a significant relationship between these traits using the same test with the same data.

Thirdly, the inability of this model to correctly predict the data may stem from the fact that it is importantly incomplete. Rajon and Masel (2011) assume that the only local selection is through the mitigation route (e.g., ASC selection), rate being modified by global trade-offs between translational fidelity and replication rate. As local selection is only available to species with high *N*_e_, they infer that mitigation (by assumption the only mode of local selection) is favored when *N*_e_ is high. However, they do not consider the case of local rate modifiers (stop codon usage), which also should respond most efficiently to selection when *N*_e_ is high. That ASC selection is not predicted by *N*_e_ but local rate modifiers are suggests that their model is incomplete (in an important manner). If so, this suggests caution in assuming veracity of downstream inferences and suggests that it is important to include local rate and mitigation (and global mitigation if this too is relevant). That we could not substantiate their extension (Meer et al. 2020) which assumed higher absolute error rates when *N*_e_ is high, causing greater TAA HEG-LEG disparity, is similarly compatible with a problem with model specification.

We suggest that extended models could quite easily explain our observations. Given that local error rates are lower when *N*_e_ is high (higher TAA usage), we might expect the lack of clear correlation with patterns of ASC enrichment (and deviation from prior predictions regarding ASC usage): When local error rates are low, selection for ASCs is low because mistakes are rare, when error rates are high this is because selection is too weak to reduce local error rate and hence selection for ASCs must also be weak. We probably need other variables, such as cellularity, to explain between-species variation in ASC enrichment.

### Remaining Conundrums

#### Why Might Selection Act More on Local Error Rates Than on Local Error Mitigation

Above we suggest a synthesis in which *N*_e_ modulates the efficiency of local selection such that error rates are lower when *N*_e_ is high which in turn dislocates any mitigation selection (ASCs). It leaves, however, several unanswered questions. Firstly, why might selection act more on local error rates than on local error mitigation? The logic of the Rajon and Masel (2011) model is that selection for local effects is associated with small selective coefficients and so most relevant in species with high *N*_e_. This renders the apparent preference for selection on rate over mitigation enigmatic. If an error, such a read-through, has a mean cost *c* per event (energy lost through translation of the 3′-UTR, deleterious protein products, etc.) and a rate *r* (proportional to the number of translation events over time), then the net cost is *c* × *r*. A mutation reducing the error rate by delta *r* (*dr*) should be associated with positive selection of strength equal to *c.dr*. Similarly, however, reducing the cost by *dc* has selection of strength *dc.r*. Given the symmetrical nature of these two, it is not at first sight obvious why selection should be focused more on the rate of error than on the cost per error. This might be because mutations affecting rate are more common than those affecting cost. This seems unlikely as rate-affecting mutations must act at or near the stop codon whereas cost can be reduced by any mutation in 3′-UTR that enables an earlier ASC.

Alternatively, the nature of the mutational effects may be different such that *c.dr* > *cd.r*, that is, reducing error rate is more visible to selection than reducing error cost. We suggest that one possible reason, at least in our system, is that TAA is so much more efficient than TGA that a TGA->TAA mutation may have an order of magnitude effect on the error rate, as evidenced in bacteria (Sambrook et al. 1967; Roth 1970; Strigini and Brickman 1973; Ryden and Isaksson 1984), but an ASC might save only a relatively small proportion of energy. For example, in AT-rich genomes there might be a stop a certain distance from the focal stop just by chance. An earlier stop codon is likely to reduce costs but not by orders of magnitude (indeed Rajon and Masel [2011] assume that any ASC renders read-through effectively neutral). In addition, ASCs may not be a perfect solution to reducing costs as they may be less effective if not within a correct context (Major et al. 2002). If so, *c.dr* ≫ *dc.r* may hold and we expect selection on local rate more than local mitigation of costs. That AT-rich genomes likely have a fail-safe stop codon by accident may also explain why we find TAA enrichment to be predicted by GC content. In GC-rich species, the costs of read-through are higher as the distance to the nearest downstream accidental stop codon is longer. Hence, the selection for TAA is stronger than when an incidental ASC is found.

If the above logic is correct, then the results derived here need not be generalizable to other error-prone systems. Although Rajon and Masel (2011) emphasize that the translational read-through system may be a generalizable exemplar (as we too indeed assumed) of error control, if the above logic is correct it would suggest that the preference for local error rate selection is largely contingent on a peculiarity of the system (high error rate variance between stops). For this reason, and contra Rajon and Masel (2011), we caution against generalizing.

If further caution against generalizing is needed, consider the case of selection on splicing. Our model for stronger TAA selection when *N*_e_ is high, and no correspondence with error mitigation, might appear to be at odds with evidence for the increased use of another local error rate modifier, ESEs, to reduce the rate of error-prone splicing in low *N*_e_ species, these having large and frequent introns (Wu and Hurst 2015). In this case, however, it is proposed that not simply are error rates higher with low *N*_e_, but they are also subject to a ratchet-like accumulation of insertions, each degrading splicing levels that bit more. As a consequence, the accumulation of many splice degrading insertions can enable selection for one exonic mutation enabling increased splice rates (hence increased ESE density, especially in proximity to large introns). In the case of stop codons, there is only one stop codon per gene so there is less possibility of an accumulation of stop codon degrading mutations. We note that the possibility that weakened local selection might itself increase error rates is not permitted in the models of Rajon and Masel (2011) or Meer et al. (2020).

#### Why Might Cellularity Matter for ASCs but Not for TAA?

A second enigma concerns the cellularity result. Although *N*_e_ does not predict ASC enrichment, that is, local error mitigation, in phylogenetically controlled tests across species, even controlling for *N*_e_, single-celled status predicts ASC enrichment. Comparison of unicell- and multicell-expressed genes within the same species provides some further, albeit marginal, support for this possibility. In *A. thaliana* and *D. discoideum*, ASC frequency immediately proximal to the primary stop is significantly higher in unicell-associated genes compared with multicell-associated genes.

We considered looking at cellularity as a variable as a priori we thought that costs of read-through errors would be different in cellular and multicellular species. The cell replacement argument, indeed, can be evoked to explain the stronger purifying selection on brain-expressed genes (Drummond and Wilke 2008), as neurons cannot be replaced following the accumulation of improperly folded protein. Why then does cellularity matter for error mitigation (ASC usage) but not for error rate (TAA usage)? Were we to have found that for TAA both *N*_e_ and cellularity matter, the logic would have been easier to discern (although there is a weak hint of this in the within-species analyses). The result is further compounded by the observation that ASC enrichment in yeasts is most pronounced in TAA-terminating genes (Liang et al. 2005), suggesting that the two processes act synergistically to safeguard HEGs.

We have no good answer to this enigma. It is possible that cellularity does not matter per se, it just happens to covary with some other variable. Indeed, across species we see a strong trend, but the within-species trend is much less robust. One possibility relates to a third parameter we have little or no access to. For example, it is known that one consequence of some prion states is greatly increased rates of translational read-through (Wickner et al. 1995; Harrison 2019). If the distribution of this problem is phylogenetically patchy, but the effect is also more acute in the unicell mode or more commonly seen in unicellular organisms, then this could go some way to explain the phenomenon.

Some sort of enigmatic phylogenetic patchiness seems to be required to explain our between-species ASC enrichment data. Although we found evidence for variable ASC enrichment among different eukaryote groups, there is also considerable unexplained intragroup variability. Indeed, in all phylogenetic groups (vertebrates, invertebrates, plants, fungi, and unicell eukaryotes) we see that some, but not the majority, of the species present genome-wide ASC enrichment. That the species with ASC enrichment in HEGs compared with LEGs tend to be those with absolute ASC enrichment underscores this enigmatic patchiness. It is possible that some genomes simply do not value ASC error mitigation and instead rely upon their efficient nonstop decay or other degradation mechanisms (Kosinski and Masel 2020). It is hence important to note that all genomes likely employ a wide range of error mitigation mechanisms (both local and global) and these may not coevolve identically in all species. Nevertheless, we considered three further possible predictors (GC content, gene size, and 3′ intergenic distance) but none was strongly predictive at genome-wide level by PGLS tests. This suggests that even allowing for cellularity and *N*_e_ there remains some very patchy predictor of ASC enrichment that we have been unable to discern.

We can support the notion of patchiness by reference to what may be happening in prokaryotes. In some bacteria, stalled ribosomes on mRNAs that do not contain a stop codon (or have had their stop codon read-through) may be rescued by alternative release factors such as ArfA (Keiler and Feaga 2014). One might predict, then, that ArfA-containing genomes have less propensity to select for fail-safe ASCs as the impact of read-through is reduced. We indeed find prima facie evidence to suggest that bacterial species with an annotated ArfA gene possess significantly lower ASC frequencies (supplementary fig. S6, Supplementary Material online). Could it be that ASCs selection is dependent on an error mitigation mechanism being missing from some eukaryotes? Understanding such possibilities and access to pan-taxon, high resolution measures of absolute read-through rates would be invaluable.

## Materials and Methods

### General Methods

All data manipulation was performed using bespoke Python 3.6 scripts. Statistical analyses and data visualizations were performed using R 3.3.3. All scripts required for replication of the described analyses can be found at https://github.com/ath32/eASCs. We acknowledge that stop codons function at the mRNA level; however, here we analyze chromosomal DNA sequences and therefore refer to the three stops as TAA, TGA, and TAG.

### Extraction and Filtering of 3′-UTR Sequences

Whole-genome sequence and gene annotation data were downloaded from Ensembl release 97 (https://www.ensembl.org/info/about/species.html, last accessed September 12, 2019) and EnsemblGenomes release 45 (http://ensemblgenomes.org, last accessed September 12, 2019). The main Ensembl set contains primarily vertebrate genomes (*n* = 216), Ensembl Metazoa contains invertebrate genomes (*n* = 77), Ensembl Plants contains plant genomes (*n* = 62), Ensembl Fungi contains fungal genomes (*n* = 1,014), and Ensembl Protists contains unicelled eukaryote genomes (*n* = 236). For all sets, genomes were filtered to retain just one genome per genus to reduce biases due to phylogenetic nonindependence that may occur due to oversampling. Species sets for each group were then manually curated to move incorrectly placed species. *Caenorhabditis elegans* and *S. cerevisiae* were removed from the vertebrates set as they are not vertebrates. Unicellular (algae) species in the plants set were removed and added to the unicellular set if not already present. Nondimorphic yeast species were removed from the fungal set and added to the unicellular set if not already present. *Candida albicans* was also added to the unicell set via bespoke download (available from www.candidagenome.org, last accessed September 12, 2019). This left a final sample of 104 vertebrates, 41 invertebrates, 22 plants, 21 fungi, and 71 unicellular eukaryotes. A full species list for each taxonomic group can be found in Source Data, Supplementary Material online.

Similar to prior analyses (Adachi and Cavalcanti 2009; Ho and Hurst 2019), for every gene in each genome a sequence inclusive of the primary stop followed by 97 nucleotides of the 3′-UTR was extracted by reference to the annotated coding sequence coordinates. Only genes with 3′ intergenic space of >100 bp were considered. Resultant sequences were filtered to retain only those 3′ sequences made up exclusively of A, T, G, and C, those from genes with one stop after the initiating codon, and those from a gene body with a nucleotide length that is a multiple of 3.

### Inferring Substitution Rate

Lists of one-to-one orthologous genes were downloaded for a diverse variety of species triplets from the appropriate Ensembl Biomart repository: 1) primates; *Homo sapiens, Macaca mulatta, Pan troglodytes*, 2) nematodes; *Caenorhabditis briggsae, Caenorhabditis remanei, and Caenorhabditis elegans*, 3) Aspergillus; *Aspergillus flavus, Aspergillus niger, Aspergillus oryzae*, 4) Drosophila; *Drosophila melanogaster, Drosophila pseudoobscura, Drosophila simulans*, and 5) Arabidopsis; *Arabidopsis halleri, Arabidopsis lyrate, Arabidopsis thaliana*. Orthologous genes were extracted from the respective genomes and filtered to retain genes with coding sequence of length 3, no premature stop codons, and stop codons TAA, TGA, or TAG. Genes from each species triplet that met our quality controls were aligned using MAFFT with the -linsi algorithm (Katoh et al. 2005). Alignments with gaps <10 codons upstream or <10 “codons” downstream were discarded from further analysis.

Mutations in coding sequence or in the immediate 3′-UTR were reconstructed using a parsimony approach as previously described (Rogozin et al. 2016; Belinky et al. 2018). As each species triplet contains two ingroups and one clear outgroup, ancestral nucleotides can be inferred for each position where the outgroup nucleotide matches that of at least one ingroup. For analysis of substitution rate, we infer mutations at all nucleotide positions from ten codons upstream to ten codons downstream of the stop for TAA-, TGA-, and TAG-terminating genes (where all three orthologs agree on the stop codon). Dividing these mutational counts by the number of valid TAA-, TGA-, and TAG-terminating genes allows the calculation of mutational frequency per site.

### Comparing Stop Codon Frequencies between HEGs and LEGs

Experimentally derived protein abundance data were downloaded for all available eukaryotic genomes from PaxDb (Wang et al. 2015). Corresponding whole-genome sequence files were downloaded from the appropriate European Molecular Biology Laboratory (EMBL) database. A list of the species included can be found in Source Data, Supplementary Material online. PaxDb external IDs and EMBL locus tags were extracted and matched to generate a sample of genomes and genes for which both PaxDb and EMBL sequence data were available for >400 genes. This filtering produced a sample of 20 eukaryotic genomes, 15 of which belong to multicellular species and 5 belong to unicells. In these genomes, genes that met our filtering criteria that feature in the top and bottom quartiles of expression were defined as HEGs and LEGs, respectively. The frequencies of each primary stop codon (TAA, TGA, and TAG) at each expression level were then calculated and compared. We calculate a standardized frequency difference (SFD) for each codon such that:

$$\mathrm{SFD}=\frac{\mathrm{HEG}\;\mathrm{frequency}-\mathrm{LEG}\;\mathrm{frequency}}{\mathrm{LEG}\;\mathrm{frequency}}.$$

### Determining Nucleotide Enrichment in HEGs

HEGs were identified and extracted as explained in the previous section. A, C, G, and T counts were counted at each site within our chosen range surrounding the primary stop codon. These counts were compared with a genome-wide null, these being the frequency of each nucleotide at the same positions in all genes regardless of expression level. Comparable “null” counts were calculated as the genome-wide frequency multiplied by the number of genes in the highly expressed set, allowing a comparison of the real observed HEG counts to the null counts using chi-square tests. Significant nucleotide enrichments or depletions were called if the chi-square tests produced a *P*-value < 0.05 (before Bonferroni correction).

### Recognition of ASC Enrichment in Multicellular Eukaryotes

As found in previous studies (Liang et al. 2005; Adachi and Cavalcanti 2009; Ho and Hurst 2019), ASC enrichment in eukaryotes is unlikely to be universally specific to one particular 3′ codon position. Hence, we repeat the methodology previously published in our assessment of ASC enrichment in unicellular eukaryotic genomes (Ho and Hurst 2019) in counting the number of genomes in each taxonomic grouping (vertebrates, invertebrates, etc.) that possess ASC enrichment (as determined by chi-square tests) at one or more site. ASCs at a particular position were considered to be enriched if they were found in raw excess to null expectation and their comparison to null produced a chi-square *P*-value below 0.05/6 (Bonferroni-corrected, ∼0.0083). Our *P*-value threshold dictates that the probability of a genome possessing no significant ASC enrichment at one or more positions by chance is (1 − 0.0083)^6^ (∼0.951). Therefore, there is a 1 − 0.951 (∼0.049) probability that a genome will contain significant enrichment at one or more positions by chance. We use this probability in a series of binomial tests to consider whether the number of genomes in our data set possessing ASC enrichment was higher, lower, or as expected due to chance. This methodology was repeated for two distinct null models: i) dinucleotide-controlled simulations (Ho and Hurst 2019) and ii) a degrading frequency null adapted from that first proposed by Adachi and Cavalcanti (2009) and used in our previous ASC analysis (Ho and Hurst 2019):

- The dinucleotide-controlled null involves the simulation of 10,000 bespoke null 3′-UTR sequences for a particular genome. Control for genome-specific dinucleotide preferences is facilitated by the capture of nucleotide and dinucleotide frequencies in a Markov-like decision process that directs nucleotide selection in the creation of each simulated sequence. ASC frequencies are calculated in the simulants for comparison with the real genome.
- The adapted Adachi and Cavalcanti (2009) (A+C) null considers only the first in-frame ASC of each UTR sequence. The null ASC frequency expectation at a given position is considered as the probability of not finding a stop at any position upstream multiplied by the probability of finding a stop at any position: First ASC probability = *p*[1 − *p*]^(^^*n*^^−1)^, where *n* is the focal codon position and *p* is the ASC frequency at any in-frame UTR position.

### Calculating an ASC Enrichment Score

To assess the relationship of ASC enrichment with any variable first requires the calculation of an enrichment score. To do this, we first calculate a positional enrichment score (PES) from positions +1 to +6 individually such that:

$$\mathrm{PES}=\frac{\mathrm{Observed}-\mathrm{Expected}}{\mathrm{Expected}},$$

where “observed” is the raw ASC count in the genome at a particular position and “expected” is the expected frequency for that position under the Adachi and Cavalcanti (2009) null hypothesis. The overall enrichment score for each genome used for the correlation analysis was the mean positional enrichment score across all positions. Scores were calculated for 24 genomes for which an existing *N*_e_ estimate was available in the literature (Gossmann et al. 2012; Lynch et al. 2016) or a bespoke *N*_e_ estimate was possible.

### Calculating a TAA Stop Codon Enrichment Score

Similar to how an ASC enrichment score is required for correlation analyses, we must calculate a variable to quantify the extent to which TAA usage is increased in a given genome. For this purpose, we calculate a TAA enrichment score such that:

$$\mathrm{TAA}\;\mathrm{enrichment}\;\mathrm{score}=\frac{\mathrm{TAA}\;\mathrm{usage}\;\mathrm{at}\;\mathrm{primary}\;\mathrm{site}-\mathrm{mean}\;\mathrm{TAA}\;\mathrm{usage}\;\mathrm{downstream}\;}{\mathrm{mean}\;\mathrm{TAA}\;\mathrm{usage}\;\mathrm{downstream}},$$

where mean TAA usage downstream is calculated from downstream codon positions +1 to +6. “Usage” refers to the relative frequency of TAA compared with the other stop codons TGA and TAG at position *n*, such that:

$$\mathrm{TAA}\;\mathrm{usage}=\frac{\mathrm{TAA}\;\mathrm{freq}.\;}{\mathrm{TAA}\;\mathrm{freq}.+\mathrm{TGA}\;\mathrm{freq}.+\mathrm{TAG}\;\mathrm{freq}.\;}.$$

### Estimation of *N*_e_

New *N*_e_ estimations were calculated using previously published species nucleotide diversity (π) and mutation rate (µ) such that:

$$\mathrm{Effective}\;\mathrm{population}\;\mathrm{size}\;\left(N_{e}\right)=\frac{\pi }{4µ}.$$

All nucleotide diversity, mutation rate, and estimated effective population size values used in this study can be found in Source Data, Supplementary Material online.

### Derivation of GC, Gene Length, and 3′ Intergenic Distance

With *N*_e_ estimated as above and cellularity considered as a binary trait (0 for multicells and 1 for unicells), we could examine the relationship between enrichment score and these two variables. In addition, GC content was calculated from all of the extracted UTR sequences of a given genome. Median gene body lengths and 3′ intergenic distances were calculated for each genome given Ensembl annotations (Source Data, Supplementary Material online).

### PGLS Analysis

Phylogenetically controlled tests were facilitated by PGLS using the caper package in R (https://CRAN.R-project.org/package=caper), with lambda (λ) predicted by maximum likelihood. Pagel’s lambda statistic (between 0 and 1) reveals the extent to which the phylogeny correctly predicts the covariance observed between species, such that λ = 0 suggests each data point is phylogenetically independent and λ = 1 suggests traits are evolving as predicted by tree topology alone. Note that adjusted *r^2^*-values reported by PGLS may be negative if the fitted model performs worse than null. The phylogenetic trees required for this analysis were generated using TimeTree (Kumar et al. 2017) and are available at https://github.com/ath32/eASCs in nexus format.

### Intraspecies Comparisons of Unicell- and Multicell-Expressed Genes

The comparison of genes associated with unicellular development to those associated with multicellular development within the same organism controls for *N*_e_ in assessing the role of cellularity in error mitigation selection. Our cellularity hypothesis predicts that unicellular-expressed genes contain more ASCs than multicellular-expressed genes. We test this prediction in two phylogenetically distinct organisms: *D. discoideum* and *A. thaliana*.

In *D. discoideum*, we compare genes associated with vegetative (unicell) growth to social (multicell) growth using data from de Oliveira et al. (2019). In their study, sociality genes were defined as those expressed >90% of the time during the sociality growth phase (>1 h following nutrient starvation). We consider any genes not included in their social genes list (available in the source data of their paper) to be associated with vegetative growth.

In *A. thaliana*, we compare genes enriched in pollen (unicell) with those depleted in pollen (multicell). To facilitate this, we acquired a list of pollen-specific and pollen-reduced genes from supplementary table 1 of Pina et al. (2005). Pollen-specific genes are those called present in pollen but absent in seedlings, leaves, siliques, and roots. Pollen-reduced genes are those expressed less often in pollen compared with other tissues.

## Supplementary Material

Supplementary data are available at *Molecular Biology and Evolution* online.

## Supplementary Material

msaa210_supplementary_data
