# Supplementary material for: Effective Population Size Predicts Local Rates but Not Local Mitigation of Read-through Errors
Source: Mol Biol Evol. 2020 Aug 14;38(1):244–62. doi: 10.1093/molbev/msaa210 (PMC7783166; doi:10.1093/molbev/msaa210)
Supplement: msaa210_supplementary_data [file msaa210_supplementary_data.pdf]

Supplementary Information – Figures followed by Tables

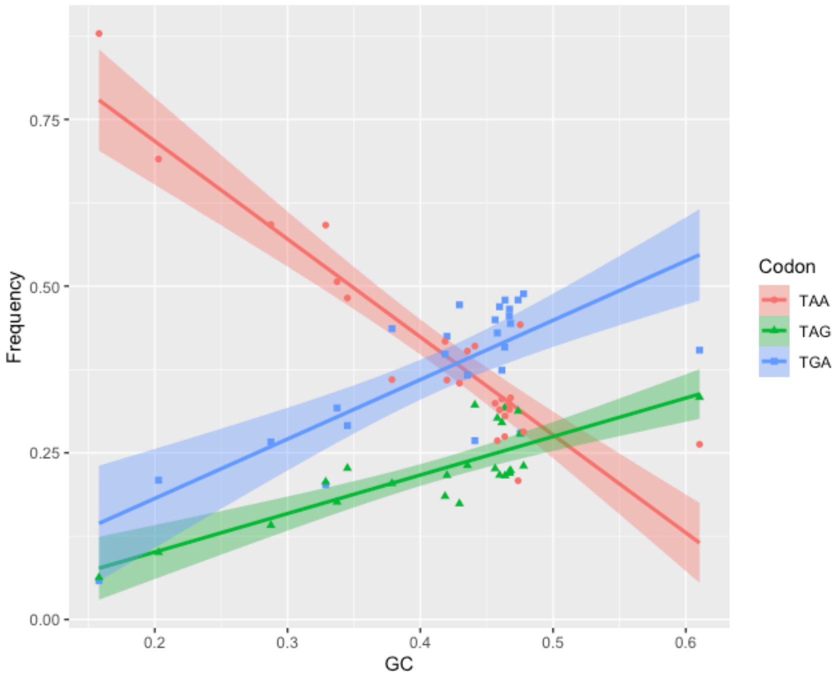

Supplementary fig. S1. Eukaryotic stop codon usage as a function of GC content calculated from the same genes of each genome. The frequencies of TAA, TGA and TAG stop codons were calculated for 25 eukaryotic genomes and assessed for a relationship with GC content. TAA frequency is negatively correlated GC content ($p = 3.2 \times 10^{-6}$, rho = -0.803; Spearman's rank), with TGA ($p = 0.00083$, rho = 0.636; Spearman's rank) and TAG ($p = 0.00012$ rho = 0.705; Spearman's rank) positively correlated. Despite its relationship with GC, TAG is always the least frequent stop codon in all cases.

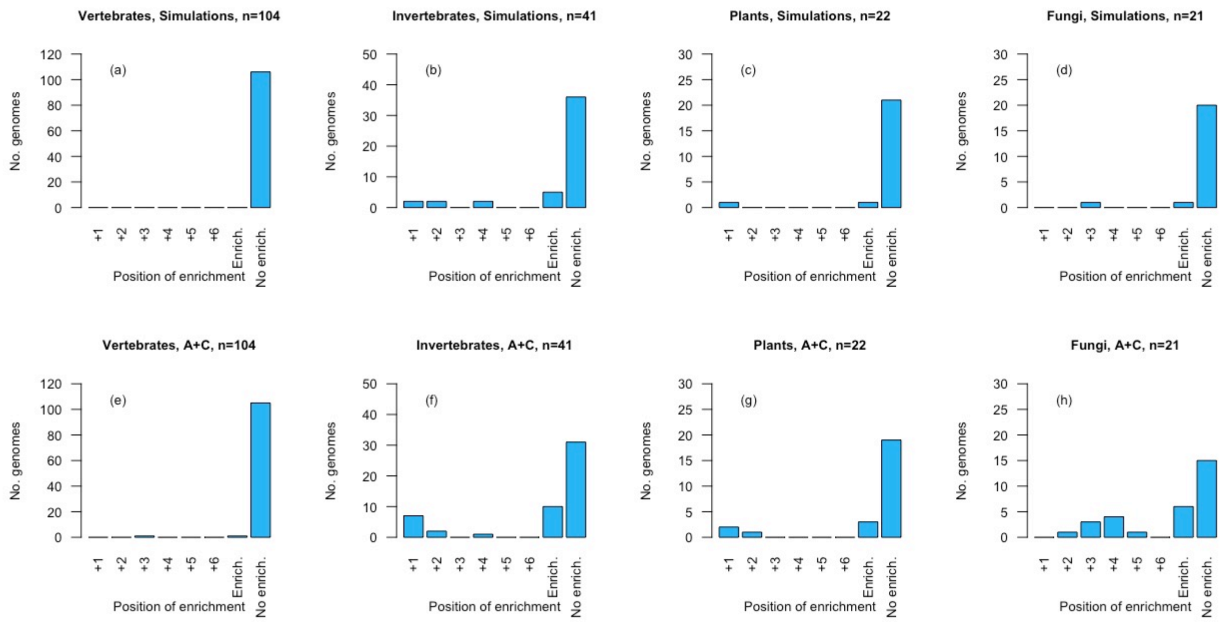

Supplementary fig. S2. Assessment of ASC enriched genomes in four multicellular eukaryote groups. ASC frequencies in each genome were compared to two null models: dinucleotide-controlled simulations (a-d) and the degraded null proposed by Adachi and Cavalcanti (e-h). In vertebrates enriched genomes are significantly under-represented against both dinucleotide-controlled simulations (0/104, p = 0.0053, one-tailed binomial test, expected 5.1) and the A+C null (1/104, p = 0.034, one-tailed binomial test, expected 5.1). In the invertebrate set enriched genomes are significantly over-represented against both dinucleotide-controlled simulants (5/41, p = 0.049, one-tailed binomial test, expected 2.0) and the A+C null (10/41, p = 2.2 x 10^{-5}, one-tailed binomial test, expected 2.0). In plants and fungi there is no significant difference in the number of enriched genomes compared to dinucleotide null (plants: 1/22, p = 1, two-tailed binomial test, expected 1.1; fungi: 1/21, p = 1, two-tailed binomial test, expected 1.0), but significant over-representation compared to the A+C null (plants: 3/22, p = 0.0091, one-tailed binomial test, expected 1.1; fungi: 6/21, p = 4.0 x 10^{-4}, one-tailed binomial test, expected 1.0).

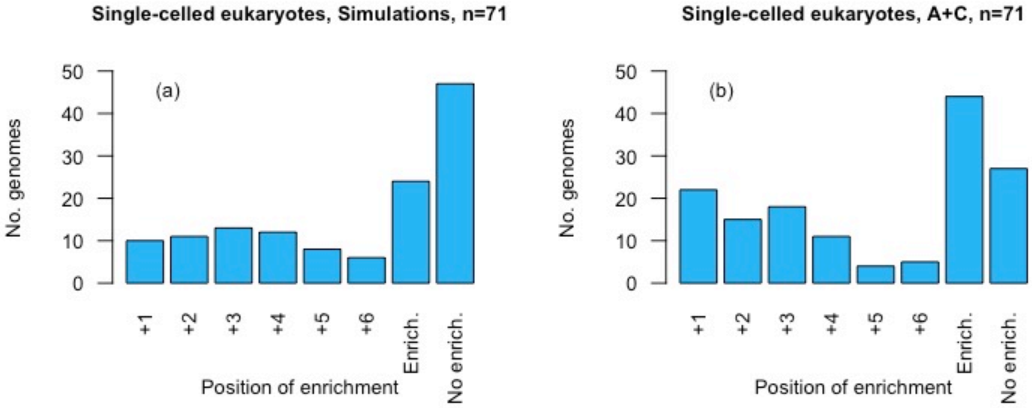

Supplementary fig. S3. Assessment of ASC enriched genomes in unicellular eukaryotes, considering position +1. ASC frequencies in each unicell genome were compared to two null models: dinucleotide-controlled simulations (a) and the degrading null proposed by Adachi and Cavalcanti (b). How do these levels of enrichment compare with those seen in multicellular eukaryotes? Despite the invertebrate set (Supplementary Figure 1) containing the largest proportions of enriched genomes (5/41 simulations and 10/41 A+C), this proportion is significantly lower ($p = 0.022$, $\chi 2 = 5.2$, Chi^2 test on dinucleotide null proportions; $p = 2.7 \times 10^{-4}$, $\chi 2 = 13.2$, Chi^2 test for A+C null proportions) than those seen in unicellular species (24/71 simulations and 44/71 A+C). This indicates that, while ASC enrichment is present and common among some multicellular eukaryotic groups, it is rarer than in unicellular organisms.

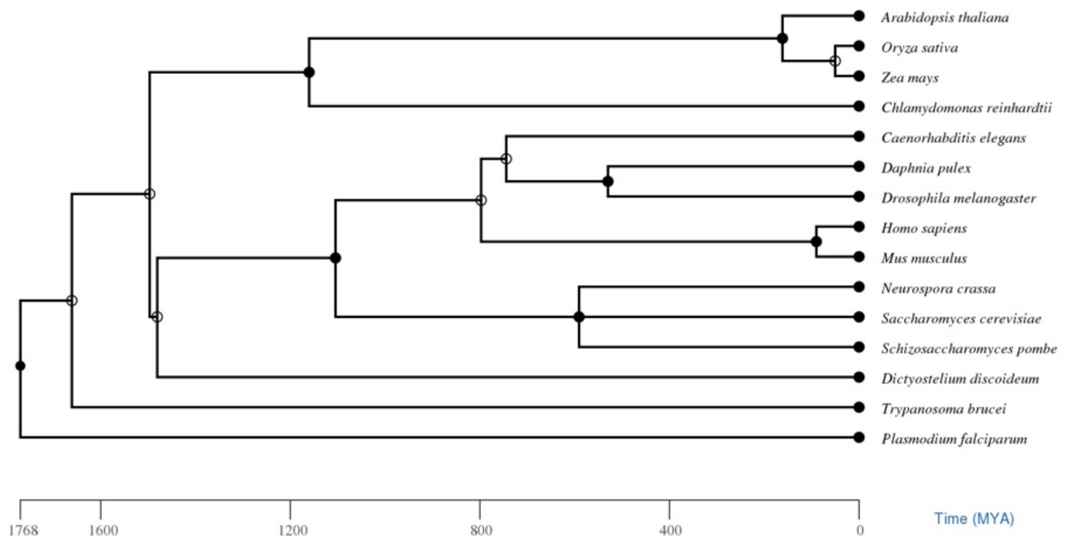

Supplementary fig. S4. Pruned phylogenetic tree describing the eukaryotic species used in PGLS analysis. 15 species were used in our phylogenetically-controlled tests for correlation, pruned from 24 species to remove species with low divergence time. The tree was derived using TimeTree, which requires a species list to be uploaded.

Supplementary Information – Figures followed by Tables

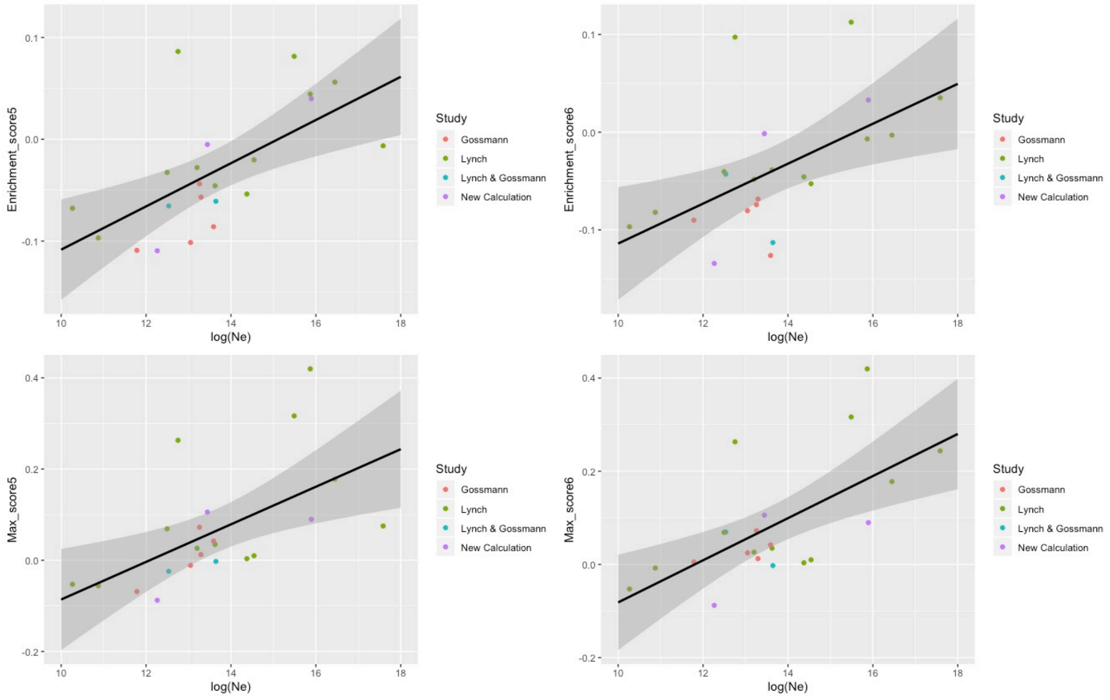

Supplementary fig. S5. Correlation analysis between N_e and four measures of ASC enrichment in 24 eukaryotes. To investigate the possibility of a relationship between N_e and ASC enrichment, we calculate ASC enrichment scores for each genome using two methods. First, we consider a score which takes the average ASC enrichment at each position (from +1 to +6 downstream; see methods of the main paper). Second, given genes possessing an ASC are unlikely to select for a third stop, we consider just the maximum score (at any position from +1 to +6). There is, however, an argument that position +1 should be ignored when considering ASC enrichment due to the possible selection of extended termination motifs immediately proximal to the primary stop. For each method, we hence calculate one score that includes position +1 and one score that excludes it. We find that all four measures of genome ASC enrichment are positively correlated with log(N_e) before Bonferroni correction (enrichment score including +1: $p = 0.0080$, enrichment score excluding +1: $p = 0.0090$, max score including +1: $p = 0.025$, max score excluding +1: $p = 0.010$).

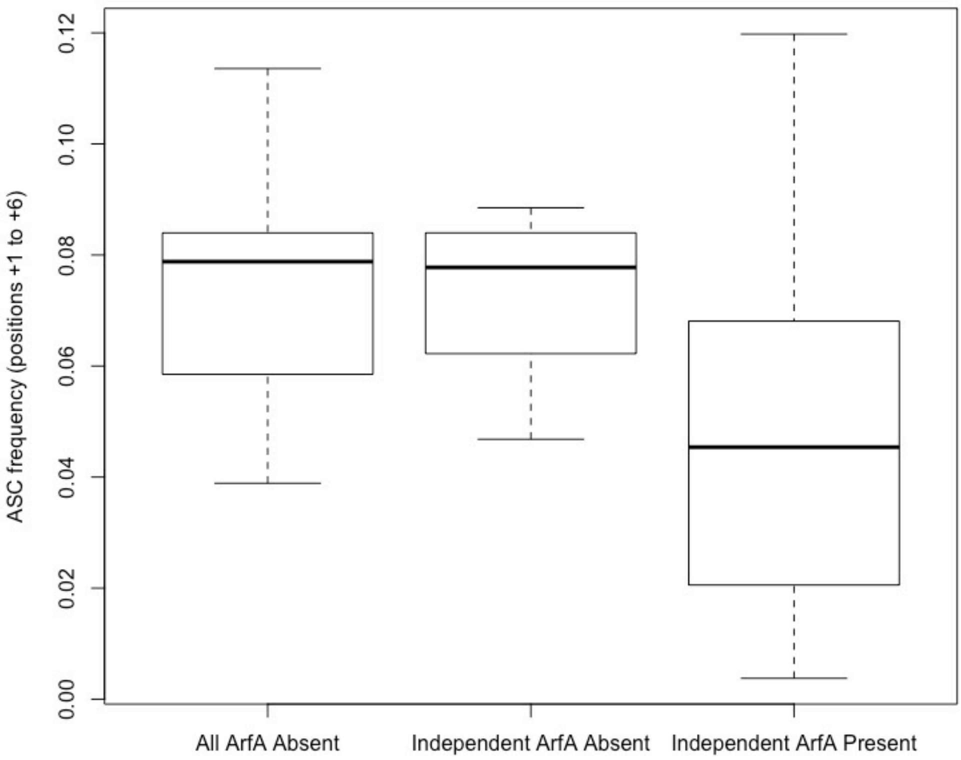

Supplementary fig. S6. Additional stop codon (ASC) frequency comparison between bacterial genomes with and without an annotated ArfA gene. ArfA is associated with ribosome rescue in mRNAs that do not contain a stop codon in bacteria, hence we predict genomes without an ArfA annotated gene to have greater selection for fail-safe ASCs. To test this prediction, we calculate ASC frequencies for all ArfA-absent genomes (n=212) available for download from EMBL, for all ArfA-absent genomes that are relatively phylogenetically independent (one genome per genus, n=6) and for similarly independent ArfA-present genomes (n = 639). Considering all ArfA-absent genomes, ASC frequencies are significantly lower than observed in the ArfA-present group ($p = 2.9 \times 10^{-15}$, Wilcoxon signed-rank test). This is corroborated when using just independent ArfA-absent species ($p = 0.0060$, Wilcoxon signed-rank test).

Supplementary Information – Figures followed by Tables

Supplementary table T1. Consensus sequences for TAA-terminating highly expressed genes in 19 eukaryotes. Nucleotides A, T, G and C are called if there exists significant enrichment ($p < 0.05$) for these bases compared to null expectations (generated from genes of all expression level in the genome) according to Chi^2 tests. A minus sign indicates significant under-enrichment compared to null. An 'N' is called is there exists no significant deviation one way or another for all bases at this position.

Species	-18	-17	-16	-15	14	-13	-12	-11	-10	-9	-8	-7	-6	-5	-4	-3	-2	-1	1	2	3	4	5	6	7	8	9	10	11	12	13	14	15	16	17	18	19	20	21
Equus_caballus	N	N	N	N	N	N	N	N	-A/C	G	N	N	N	N	N	N	N	N	T	A	A	N	N	-A/T	N	N	N	N	N	N	N	N	N	N	-T/G	-T	N	G	N
Apis_mellifera	N	N	N	N	N	N	N	N	N	N	N	N	N	N	N	N	N	N	T	A	A	N	N	N	N	N	N	N	N	N	N	N	N	N	N	N	N	N	N
Trypanosoma_brucei	-T	N	G	N	N	-T	-T	A/-T	C	G	-T/C	N	N	A/-T	-A/G	N	-T	C	T	A	A	G/-C	N	-T	N	N	N	N	N	N	G	N	N	N	N	N	N	N	N
Chlamydomonas_reinhardtii	A/-G	N	N	N	A/-C	N	-A/T/G/-C	G	-G	T/-C	-G	N	-C	-A	N	G	-A/G	T/-G/C	T	A	A	N	N	-A/G	-G/C	-G	N	T/-G	N	T	N	T	N	C	T	N	N	N	N
Homo_sapiens	N	N	N	N	N	N	N	-A/C	N	N	N	N	N	N	G	N	N	N	T	A	A	N	N	-C	N	C	N	N	N	N	N	N	N	T/-C	N	N	N	N	N
Bos_taurus	N	N	-A	-A/C	C	-A	N	A/-T	N	-T	N	N	N	-T	N	-T	N	-T	T	A	A	-A/C	C	A	-T	N	N	-A/C	N	N	-A/-T/G	N	-A	N	A	-T/G	N	N	-A
Dictyostelium_discoideum	N	N	N	N	N	N	N	N	N	N	N	N	N	-T	N	N	N	N	T	A	A	N	N	N	N	N	N	-C	N	N	N	N	N	N	N	N	-G	N	N
Gallus_gallus	N	N	N	N	A/-T/-G	A/-T	N	N	G/-C	A/-T/-C	N	N	A/-C	A/-C	A/-C	N	A/-G	N	T	A	A	N	-G	-G	N	-A/T	N	N	N	N	N	-G	N	-T/G	-T	N	N	-C	N
Saccharomyces_cerevisiae	-A/G	C	N	-T/G	N	T	N	N	N	G/-C	N	C	G/-C	A/-T	N	G	A/-G	N	T	A	A	G/-C	N	N	N	-T	N	T	A/-T	N	N	A	N	N	N	N	N	N	N
Oryza_sativa	N	-G	N	N	N	N	-C	-G/C	-A/C	N	-T	N	-T	N	N	N	A/-T/-G	-T	T	A	A	N	N	N	N	N	N	N	N	N	C	N	N	N	N	-T	N	N	N
Xenopus_tropicalis	N	-G	N	N	-G	N	-T	N	N	G/-C	N	N	G	N	C	N	N	N	T	A	A	A/-C	C	N	-A/G	N	T	N	N	-A/T	N	N	N	N	N	N	N	N	N
Plasmodium_falciparum	N	N	N	N	N	N	N	N	N	-T	N	N	N	N	N	-A	N	C	T	A	A	N	N	N	N	N	N	N	N	A/-T	N	N	N	N	N	N	N	-T	N
Arabidopsis_thaliana	N	N	N	N	N	N	N	N	N	N	N	N	N	N	N	N	N	N	T	A	A	N	N	N	N	N	N	N	N	N	N	N	N	N	N	N	N	N	N
Anopheles_gambiae	-T	C	-A/-T/G/C	N	N	-A/-T/G/C	-T/G	-T	-A/C	G	A	-A/C	N	-T	-A/G/C	G	A/-G	-A/-T/C	T	A	A	-T/G/-C	-G/C	C	G	-A/T	-A	N	N	C	N	N	C	N	-A/G	-A	C	N	N
Rattus_norvegicus	C	N	N	N	N	N	N	N	-A/G/C	G	N	N	-A/G	N	N	N	N	N	T	A	A	N	N	N	N	-A	N	C	N	N	N	N	C	N	N	N	N	N	N
Schizosaccharomyces_pombe	N	N	N	N	N	N	N	-G	N	N	N	N	N	N	N	N	N	N	T	A	A	N	N	N	N	N	N	N	N	N	N	N	N	N	N	-A	N	N	N
Aspergillus_niger	N	N	N	N	N	N	N	N	N	N	N	N	-A/T	C	-T/C	N	N	-T	T	A	A	N	N	N	N	N	N	N	N	N	N	A/-G	N	N	N	-C	A	-T	N
Danio_rerio	N	-G	-A/C	G	N	N	G/-C	N	-T	N	A/-C	-A/G	-A	A/-T	-A/C	N	A	-T/C	T	A	A	A/-T/-C	C	N	N	N	-A	A	N	N	N	N	N	N	N	-G	N	-A/C	N
Caenorhabditis_elegans	N	N	C	N	N	C	G	G	C	G/-C	A/-T	C	N	N	N	G	-T/C	N	T	A	A	-C	-A	N	N	N	-A	N	C	C	N	N	N	N	N	C	N	N	N

Supplementary table T2. Consensus sequences for TGA-terminating highly expressed genes in 19 eukaryotes. Nucleotides A, T, G and C are called if there exists significant enrichment ($p < 0.05$) for these bases compared to null expectations (generated from genes of all expression level in the genome) according to Chi^2 tests. A minus sign indicates significant under-enrichment compared to null. An 'N' is called is there exists no significant deviation one way or another for all bases at this position.

Species	-18	-17	-16	-15	14	-13	-12	-11	-10	-9	-8	-7	-6	-5	-4	-3	-2	-1	1	2	3	4	5	6	7	8	9	10	11	12	13	14	15	16	17	18	19	20	21
Equus_caballus	N	N	N	N	N	N	-T/C	N	N	N	N	-T/G	N	N	N	T	A	N	T	G	A	N	N	N	N	N	-G/C	N	N	N	C	C	A	N	N	N	N	N	N
Apis_mellifera	N	N	N	C	N	N	N	N	N	N	-G	N	N	N	N	N	-A	N	T	G	A	N	N	N	N	N	N	N	N	N	N	N	N	N	N	N	N	N	N
Trypanosoma_brucei	A/-T	N	N	N	N	-T	N	N	N	N	N	N	-T/C	A/-T	-T	N	A	N	T	G	A	G/-C	G	N	N	N	N	N	N	-T/G	N	G	N	N	N	A/-T	-C	N	N
Chlamydomonas_reinhardtii	N	-G	-A	N	N	N	N	N	N	N	N	-A	C	N	N	N	N	N	T	G	A	N	N	N	N	N	N	N	N	N	G	N	N	N	N	N	-A	N	N
Homo_sapiens	N	N	N	N	N	N	N	N	N	N	N	N	N	N	N	N	-C	C	T	G	A	N	A	N	N	N	N	G	N	N	N	N	N	N	N	N	N	N	N
Bos_taurus	N	N	N	-T	-C	-A	-A/C	C	-A/C	G	G	N	N	A	N	C	A/-C	N	T	G	A	N	N	-A/T	-A/G	N	-T	N	N	-A/C	N	N	-A	-T/G	N	-A/C	-G	N	-T
Dictyostelium_discoideum	N	N	N	N	N	N	N	N	N	N	-C	N	N	N	N	G	N	N	T	G	A	N	N	N	N	N	N	N	N	N	N	N	N	N	N	N	N	N	N
Gallus_gallus	A/-C	N	N	A	N	N	N	N	-T	N	G	N	N	N	N	N	A	N	T	G	A	A/-C	N	N	N	T/-C	N	-T	-G	N	T/-G	N	N	N	N	N	N	N	N
Saccharomyces_cerevisiae	-C	N	-G	-C	N	N	G	C	N	N	A	N	N	N	N	N	N	N	T	G	A	T/-C	-A	N	N	N	-A	N	N	N	N	N	N	N	N	T	N	N	N
Oryza_sativa	N	C	N	N	N	N	N	N	-T/C	N	N	C	N	N	N	A	N	N	T	G	A	N	G	N	N	G	N	N	N	N	N	N	N	N	N	N	-C	N	N
Xenopus_tropicalis	C	N	C	N	N	N	N	G	N	-A/C	N	N	G	G	G	N	G	N	T	G	A	A	T	N	T	N	N	T	N	N	N	N	N	N	N	N	N	N	N
Plasmodium_falciparum	N	N	N	N	N	N	-T	N	N	N	N	N	N	N	-G	N	N	N	T	G	A	N	N	N	N	N	N	N	N	N	N	N	N	N	N	N	N	N	N
Arabidopsis_thaliana	N	N	A	N	N	N	N	N	N	N	N	T	N	N	N	N	N	N	T	G	A	N	N	N	N	N	N	N	N	N	N	N	N	N	T	N	N	N	N
Anopheles_gambiae	N	N	N	N	-G	N	N	N	N	N	N	N	N	N	N	A/-G	A/-T	-A/C	T	G	A	N	-T	N	N	N	C	N	-T	N	N	N	N	N	N	N	N	N	N
Rattus_norvegicus	N	N	-A	N	N	N	G	N	N	N	N	N	N	N	N	-T/C	-G	-A/-T/G	T	G	A	N	N	N	N	N	-A	N	N	N	N	-A/C	N	N	N	N	N	C	C
Schizosaccharomyces_pombe	N	N	N	N	N	N	N	N	N	N	N	N	N	N	N	N	N	N	T	G	A	N	N	N	N	N	N	N	N	N	N	N	N	N	N	N	N	N	N
Aspergillus_niger	N	N	N	N	-C	N	N	N	N	N	N	N	N	N	N	N	N	N	T	G	A	N	N	N	N	N	N	N	N	N	N	N	N	N	N	N	N	A	N
Danio_rerio	N	N	G	G	N	A/-C	N	N	N	-T	N	N	N	N	G	G	A	N	T	G	A	-C	N	N	-A/C	N	-A	N	C	-G	T	N	-A/C	N	N	N	N	N	N
Caenorhabditis_elegans	N	N	N	N	N	G	N	A	N	N	N	C	N	-T	N	N	N	-T/C	T	G	A	-C	-G	N	C	C	N	N	N	N	N	N	N	N	N	N	C	N	-A/T

Supplementary Information – Figures followed by Tables

Supplementary table T3. Consensus sequences for TAG-terminating highly expressed genes in 19 eukaryotes. Nucleotides A, T, G and C are called if there exists significant enrichment ($p < 0.05$) for these bases compared to null expectations (generated from genes of all expression level in the genome) according to Chi^2 tests. A minus sign indicates significant under-enrichment compared to null. An ‘N’ is called is there exists no significant deviation one way or another for all bases at this position.

Species	-18	-17	-16	-15	14	-13	-12	-11	-10	-9	-8	-7	-6	-5	-4	-3	-2	-1	1	2	3	4	5	6	7	8	9	10	11	12	13	14	15	16	17	18	19	20	21
Equus_caballus	N	N	N	N	N	N	N	N	N	N	N	N	N	N	N	N	N	N	T	A	G	N	N	G	N	N	N	N	N	N	N	N	C	N	N	N	N	N	N
Apis_mellifera	N	N	N	N	-C	N	N	N	N	N	N	N	N	N	N	N	N	N	T	A	G	-T	N	N	N	N	N	N	N	N	N	N	N	-G/C	N	N	N	N	N
Trypanosoma_brucei	N	A	G	N	N	G	A	N	-A/G	N	N	N	-T	N	-A	N	A/-G/-C	-T/C	T	A	G	A/-C	N	N	N	N	N	N	N	N	N	N	N	N	N	N	N	-C	N
Chlamydomonas_reinhardtii	N	N	N	N	N	N	N	N	A/-C	-A	N	N	N	N	N	N	N	N	T	A	G	N	N	N	N	N	C	A	N	N	N	N	N	N	N	N	N	N	-G/C
Homo_sapiens	N	N	N	N	N	N	N	N	N	N	N	N	N	N	A	N	N	N	T	A	G	N	N	-G	N	N	N	N	N	N	N	N	N	N	N	N	N	N	N
Bos_taurus	N	-G	-A/-T/C	N	-G	N	N	N	N	-G/C	N	N	N	N	N	N	N	-C	T	A	G	N	A	N	-G/C	-G/C	-G	N	N	T	T	T/-G	N	T	-A/T	-T	T	N	N
Dictyostelium_discoideum	N	N	N	N	N	N	N	N	N	N	A/-C	N	N	N	N	N	N	N	T	A	G	N	N	N	N	N	N	N	N	N	A	N	N	N	A	N	N	N	N
Gallus_gallus	A/-C	N	N	N	-G	N	-A	N	N	-C	A	A	N	N	N	T	A/-C	N	T	A	G	N	-G	-A	A/-C	N	-T	A	N	-A	N	N	N	N	N	A/-G/-C	-T	N	-C
Saccharomyces_cerevisiae	G/-C	-T	N	-T/G	N	N	G	N	N	-C	A/-T	N	N	N	N	G	A/-T/-G	N	T	A	G	A	N	N	N	N	T/-C	-A	N	N	N	N	N	N	N	N	N	N	N
Oryza_sativa	N	A	N	N	N	N	N	N	N	N	N	C	N	-A	N	N	N	N	T	A	G	N	-T/G	N	N	N	N	N	N	-A	N	N	-A/C	-A/T	N	N	T	N	N
Xenopus_tropicalis	N	N	N	A	N	N	N	N	A	N	N	N	N	N	N	N	N	N	T	A	G	N	N	N	N	N	N	N	N	G	N	N	N	N	N	N	N	N	N
Plasmodium_falciparum	N	-T	-A/T	N	-A/T	N	-T	N	N	-T/G	-T	N	N	N	N	N	N	N	T	A	G	N	N	N	A/-T	-G	N	A	A/-G	N	-G	-T	-T	N	-G	A/-G	N	N	A
Arabidopsis_thaliana	G	N	N	N	N	N	-C	N	-A	N	N	N	N	N	N	N	N	N	T	A	G	N	N	N	N	-G	N	N	N	N	N	N	N	N	N	N	N	N	N
Anopheles_gambiae	N	-T/C	N	N	N	N	N	N	N	N	N	-A	N	N	-A/C	N	A	N	T	A	G	A/-T/-C	N	A	G	-A	N	-T/C	N	N	N	N	N	N	N	N	N	N	N
Rattus_norvegicus	N	A	C	A/-C	N	N	-A	N	N	G	N	G	G	N	N	N	N	N	T	A	G	N	N	N	N	N	N	N	N	N	N	N	N	N	-T	N	N	N	N
Schizosaccharomyces_pombe	N	N	N	N	N	A	N	N	N	N	N	N	N	N	N	N	N	N	T	A	G	N	N	N	N	N	N	N	N	N	N	N	N	N	N	-G	N	N	N
Aspergillus_niger	N	N	N	N	A	N	N	N	N	N	N	N	N	N	N	N	N	N	T	A	G	N	N	N	A	G	N	N	N	N	N	-C	N	N	N	N	-T	N	N
Danio_rerio	N	T	-T/G	-C	N	N	-C	N	N	N	N	N	N	G	-A	N	A	-A	T	A	G	N	N	N	N	N	N	N	N	N	N	N	A	N	N	N	N	N	N
Caenorhabditis_elegans	N	-T	N	N	N	C	N	N	N	N	A/-T	N	N	N	N	N	A	N	T	A	G	N	N	N	N	N	N	N	N	N	N	N	N	N	N	N	N	N	N
